# Leveraging Artificial Intelligence and Large Language Models for Cancer Immunotherapy

**DOI:** 10.1002/advs.202521936

**Published:** 2026-02-25

**Authors:** Xinchao Wu, Mengtao Sun, Lusheng Li, Jieqiong Wang, Shibiao Wan

**Affiliations:** ^1^ Department of Genetics, Cell Biology, and Anatomy University of Nebraska Medical Center Omaha Nebraska USA; ^2^ Department of Neurological Sciences University of Nebraska Medical Center Omaha Nebraska USA; ^3^ Fred and Pamela Buffett Cancer Center University of Nebraska Medical Center Omaha Nebraska USA

**Keywords:** artificial intelligence, cancer immunotherapy, foundation model, machine learning, multi‐omics

## Abstract

Cancer immunotherapy, which leverages the immune system to combat tumor cells, has made significant advancements in oncology treatment in recent years. Yet significant challenges remain in predicting treatment responses and understanding mechanisms of resistance. Artificial intelligence (AI) and machine learning (ML) provide powerful tools to address these challenges, enabling breakthroughs in patient stratification, biomarker discovery, and treatment strategy optimization. While remarkable progress has been made in developing deep learning frameworks, including large language models (LLMs) to integrate the exponentially growing multi‐omics biomedical data for cancer immunotherapy, little effort has been made to systematically and comprehensively summarize these developments or critically evaluate their translational potential. To fill these gaps, this review comprehensively examines the current landscape and future directions of AI/ML applications in cancer immunotherapy. Specifically, we discuss four key areas in AI for cancer immunotherapy: (1) patient stratification, (2) biomarker discovery, (3) treatment strategy optimization, and (4) foundation models and LLMs for cancer immunotherapy. In addition, we also critically discuss current limitations and future directions for existing AI approaches for cancer immunotherapy, highlighting the actionable insights and roadmaps to accelerate the integration of AI/ML into precision cancer immunotherapy.

## Graphical abstract

Cancer immunotherapy faces challenges in predicting treatment responses and understanding resistance mechanisms. Artificial intelligence (AI) and machine learning (ML) offer powerful solutions for cancer immunotherapy in patient stratification, biomarker discovery, treatment strategy optimization, and foundation model development. This review comprehensively examines AI/ML applications across these key areas and critically discusses existing limitations and future directions for AI‐based cancer immunotherapy.

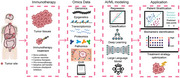

## 1 Introduction

Cancer immunotherapy, which is to mobilize and leverage the host immune system against malignant cells, has profoundly transformed oncology over the past decade, delivering remarkable remissions across diverse hematological malignancies and solid tumors [1]. For instance, immune‐checkpoint blockade (ICB) [2] and adoptive cell therapies [3] have delivered durable remission across a remarkably diverse range of malignancies. It can induce long‐term survival in metastatic melanoma [4], non‐small cell lung cancer (NSCLC) [5], renal‐cell carcinoma (RCC) [6], and hepatocellular carcinoma (HCC) [7]. Moreover, some ICB treatment plus chemotherapy extends overall survival in triple‐negative breast cancer [8]. Yet due to tumor heterogeneity and immune microenvironment variability, significant challenges remain in identifying which patients will respond to immunotherapy and understanding the mechanisms driving treatment resistance, complicating the accurate molecular diagnosis of tumor subtypes and immune responsiveness [9, 10]. Most solid tumors harbor spatially distinct clones with variable neoantigen load, MHC expression, and cytokine milieus, creating patchy sensitivity to immune attack [11]. In addition, for dense stroma, myeloid‐derived suppressor cells and T‐regulatory cells often create an immunosuppressive microenvironment [12], further dampening immunotherapy treatment efficacy. These complexities also thwart rational combination design, while their mechanistic interplay and overlapping toxicities make exhaustive clinical testing impossible [13].

In recent years, a range of immunotherapeutic modalities have been developed, each targeting different aspects of the tumor‐immune interaction. Cancer immunotherapy now encompasses five major modalities, namely (i) immune‐checkpoint inhibitors (ICIs), which block inhibitory pathways to enhance T cell activity; (ii) engineered cell therapies such as chimeric antigen receptor (CAR)‐T/natural killer (NK) cells; (iii) monoclonal and bispecific antibodies targeting tumor antigens; (iv) oncolytic viruses that selectively infect and kill tumor cells; and (v) personalized neoantigen vaccines designed to elicit targeted immune responses [14]. ICIs can deliver long‐term remission in melanoma [15], NSCLC [16], and renal‐cell carcinoma [17], but fewer than one‐third of patients with pancreatic or prostate cancer respond, largely because T cells fail to infiltrate these “cold” tumors [18]. CAR‐T cells achieve >80% complete responses in B‐cell malignancies [19], yet their efficacy against solid tumors is hampered by poor trafficking, antigen heterogeneity, and immune‐suppressive stroma [18]. Monoclonal antibodies offer exquisite target specificity but can be undermined by high production costs and on‐target/off‐tumor toxicities [20]. Oncolytic viruses promise breadth of neoantigen coverage, yet manufacturing logistics and inconsistent immunogenicity have slowed broad adoption [21].

To overcome the challenges posed by tumor heterogeneity and immune resistance, advanced omics profiling techniques have emerged as powerful tools to characterize the tumor microenvironment (TME) and guide immunotherapy. For instance, RNA‐sequencing (RNA‐seq) could quantify immuno‐inflamed signatures beneficial to ICI treatment [22]. Assay for Transposase‐Accessible Chromatin with sequencing (ATAC‐seq), which assesses the chromatin accessibility of the patients, provides in‐depth epigenetic profiling showing the regulators associated with immunotherapy agents [23]. Spatial transcriptomics could help to illustrate the spatial relationships between immune hot spots with tumors, discriminating the immune‐hot niches, which is vital to immunotherapy efficacy [24]. Proteomics validates the cell surface antigens, serving as hubs in ICI treatment or ligands for CAR‐T and bispecific antibody therapy [25]. Additionally, single‐cell techniques can yield millions of feature‐rich observations, which ultimately dissect the tumor micro‐environment, supporting ground‐breaking biomarker discovery, including the signatures of gene expression, rare cell populations, and signaling pathways important to the effectiveness of the immune system [26]. Collectively, these assays turn previously opaque resistance mechanisms into actionable biomarkers, paving the way for a more profound understanding of the intricacy of tumor heterogeneity. However, the tons of data generated from these multi‐omics assays make it difficult to interpret. These datasets often contain non‐linear interactions between genes and pathways, batch effects from different experimental conditions, and high dimensionality that complicates statistical modeling and increases the risk of overfitting [27]. To address these challenges, artificial intelligence (AI) and machine learning (ML) are emerging as powerful computational tools for cancer immunotherapy, enabling breakthroughs in patient stratification, biomarker discovery, and treatment strategy optimization. AI‐guided in‐silico trials and mathematical modeling are beginning to prioritize schedules, yet prospective validation remains scarce [28].

Over the past two decades, various AI/ML frameworks have been extensively applied to different aspects of biological studies. For instance, graph neural networks (GNNs) have been applied to model cell‐cell communication [29], variational auto‐encoders (VAEs) have facilitated multi‐omics data integration [30], and latent space alignment techniques have enabled multi‐batch harmonization [31]. These approaches help unify heterogeneous data sources and extract composite biomarkers that characterize the TME. Building upon these foundations, the application of AI/ML techniques to cancer immunotherapy presents unique challenges and opportunities. Unlike life science applications, cancer immunotherapy demands that computational models to be tightly aligned with the immune‐oncology cycle, capturing the dynamic interplay between tumor cells, immune cells, and the microenvironment to enable truly personalized therapeutic strategies [32]. There are diverse tasks in cancer immunotherapy that rely on an AI/ML framework to deal with, including patient stratification, biomarker identification, therapeutic‐design optimization, and the most challenging foundation models [33]. Reliable patient stratification enables doctors to select the best‐matched treatment, diminishing the cost and indispensable therapy [34]. Biomarkers, whether molecular, cellular, or imaging‐based pathologic, play a critical role in identifying tumor molecular subtypes [35] and guiding therapeutic decisions. While monotherapy may be insufficient for some patients, AI can support the design of synergistic treatment combinations to enhance efficacy [36]. To fully understand immune‐suppressive complex and immunity resistance within the microenvironment, AI enables the integration of the latent space of intricate multi‐omics data to dissect the multi‐omics signatures of the immunosuppressive innate and genomics‐to‐phenotypic governing mechanism resulting in the immune system against of tumorigenesis [37]. Most recently, foundation models have been introduced into the fields of computational biological studies [38]. It exploited massive biological big data through modern large‐scale deep learning (e.g., transformer models) to enable diverse downstream tasks. These tasks could be directly adapted to the fields related to cancer immunotherapy. Although AI/ML frameworks have advanced rapidly in cancer immunotherapy, so far little effort has been made to comprehensively and systematically review these developments or rigorously assess their potential for clinical applications.

Multimodal fusion is increasingly central to immunotherapy modeling because clinical variables, imaging/digital pathology, and omics each capture complementary facets of immune context. In practice, fusion strategies can be grouped by where integration occurs. Early fusion concatenates features at input and trains a single model, which is simple and effective when modalities are consistently measured and well‐harmonized, but fragile to missingness, batch effects, and cross‐site distribution shift. Intermediate fusion learns modality‐specific encoders and integrates representations via interaction‐aware modules, improving mechanistic interpretability and enabling explicit cross‐modal reasoning, yet increasing architectural complexity and overfitting risk in the small‐to‐moderate cohorts typical of immunotherapy studies unless regularization and external validation are stringent. Late fusion combines unimodal predictions, which often best match real‐world deployment because it tolerates partial modality availability and allows modular updates as assays evolve. However, it may underuse fine‐grained inter‐modal dependencies and can be capped by the weakest unimodal component. Consequently, fusion choice is context dependent. Early fusion favors uniform datasets, intermediate fusion suits carefully matched multimodal cohorts where cross‐modal biology is a primary objective, and late fusion is typically most robust under heterogeneous clinical reality.

For a self‐contained methodological foundation, two widely used architectural families are particularly relevant. GNNs extend deep learning to relational biomedical data by iteratively updating node embeddings through neighborhood message passing, thereby capturing local and higher‐order interaction structure; this maps naturally to cell–cell communication graphs, gene regulatory networks, and spatial neighborhoods. Large language models (LLMs) are transformer‐based DL models trained on large‐scale data. Transformers operate on sets or sequences of tokens (e.g., text units, image patches, or omics feature elements) and use multi‐head self‐attention to learn long‐range, data‐adaptive dependencies without hard locality assumptions. And it applies positional encodings, then preserves structures such as spatial arrangement or feature identity. These properties make transformers attractive as general‐purpose backbones for multimodal immunotherapy modeling, where clinically meaningful signals may be distributed across distant features and modalities.

Recent work in cancer immunotherapy increasingly positions AI as a discovery engine that can generate mechanistic hypotheses and drive “lab‐in‐the‐loop” iteration, rather than just a predictive machine. First, foundation and generative models trained on large single‐cell/spatial datasets can learn latent programs of myeloid polarization and niche‐specific signaling [39]. This property makes it practical to propose candidate regulators of response and mechanistic hypotheses about how the tumor‐immune ecosystem rewires under checkpoint blockade or cell therapy. However, recent evaluations also show that popular single‐cell foundation models can have meaningful generalization limitations [40], reinforcing the role as hypothesis generation rather than definitive mechanistic proof. In parallel, perturbation‐aware models and standardized benchmarking efforts are maturing around single‐cell CRISPR screens [41]. These models enable counterfactual in silico perturbation to prioritize causal regulators, synthetic‐lethal immunomodulatory targets, and rational combination strategies for cancer immunotherapy. Finally, LLM‐enabled literature agents are emerging as practical “scientific copilots” for immunotherapy discovery [42], in which they can retrieve and reconcile evidence across checkpoints, cytokines, tumor‐intrinsic pathways, and clinical trial outcomes, surface contradictions, and output experiment‐ready hypotheses. The multi‐LLM frameworks proposed specifically for immunotherapy for immunotherapy biomarker prioritization and broader agentic systems for drug discovery that are adaptable to immuno‐oncology [43]. A likely near‐term practice is that immunotherapy labs will adopt a closed‐loop discovery pipeline, which foundation models nominate immune programs and candidate control points, perturbation models triage interventions and combinations in silico; LLM agents generate literature‐grounded rationales, and focused validation tests a smaller, higher‐value set of hypotheses.

**FIGURE 1:**
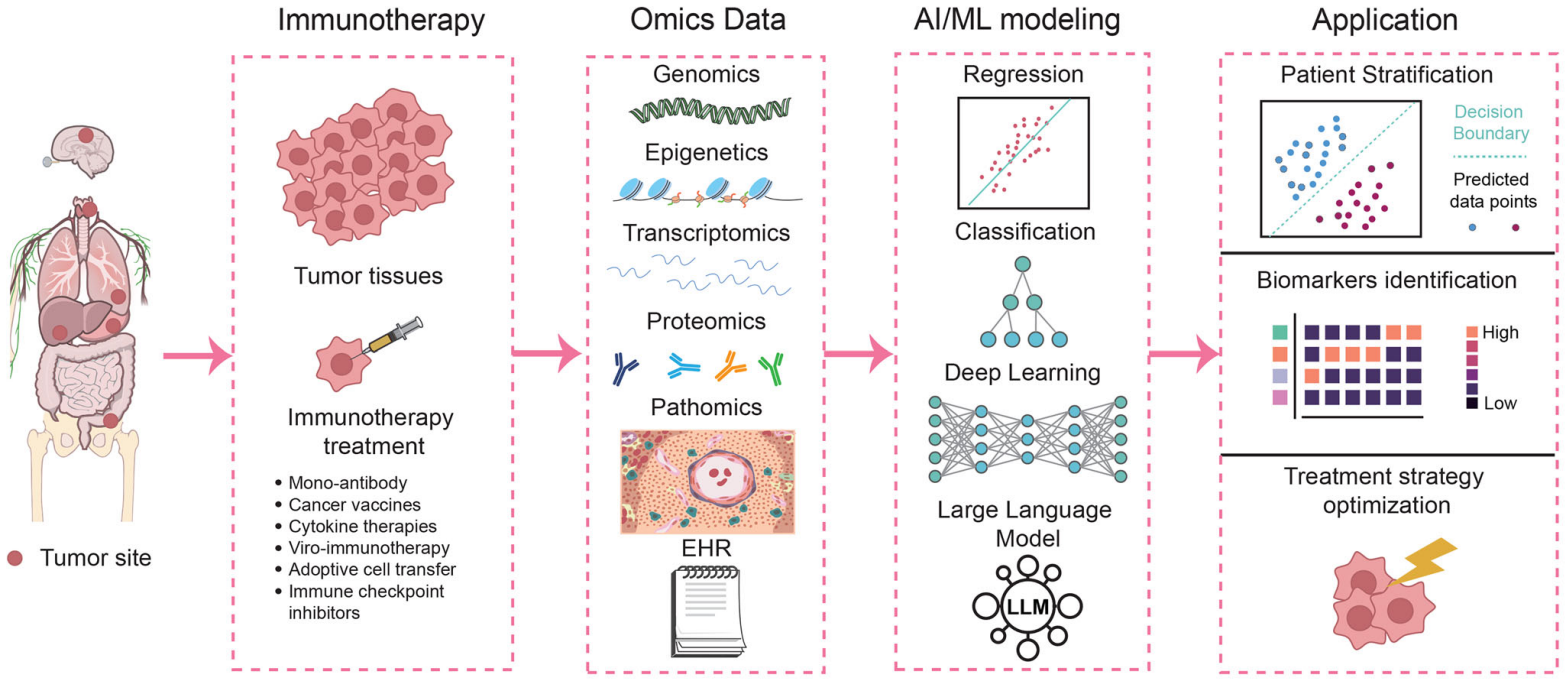
An overview of AI/ML models applied for cancer immunotherapy. Human tissues or organs, such as the brain, throat, liver, lung, stomach, and colon, represent potential sites for cancer development. Tumors originating from these regions demonstrate considerable heterogeneity for their microenvironment, dynamic immune interactions, and specific pro‐tumor mechanisms, complicating effective therapeutic interventions. Cancer immunotherapy strategies, including monoclonal antibodies, cancer vaccines, cytokine therapies, viro‐immunotherapy, adoptive cell transfer, and ICI, can substantially improve patient prognosis when supported by precise diagnostics. To precisely improve the efficacy of cancer immunotherapy, comprehensive omics data such as genomics, epigenetics, transcriptomics, proteomics, pathomics, and electronic health record data are collected. AI and ML methodologies, including regression models, classification models, deep learning, and large language models, leverage these extensive datasets for clinical decision making. Key applications include patient stratification, where AI‐driven decision boundaries categorize patient groups based on multi‐dimensional data; biomarker identification, involving predictive biomarkers such as genes, mutations, or pathological features; and treatment strategy optimization, exemplified by decisions regarding the incorporation of additional therapeutic protocols.

To fill these gaps, this review comprehensively examines the current landscape and future directions of AI/ML applications in cancer immunotherapy. Specifically, an overview of leveraging AI/ML for cancer immunotherapy is summarized in Figure 1. We explore AI/ML applications across diverse tumor types and immunotherapeutic modalities. These applications leverage multi‐ modal scale data sources, including genomics, transcriptomics, proteomics, pathomics, and electronic health records (EHR). The AI/ML approaches can be leveraged from conventional regression and classification methods to advanced deep learning architectures and foundation models, including large language models (LLMs). Specifically, we discuss four key areas where AI/ML frameworks are transforming cancer immunotherapy: (1) patient stratification, (2) biomarker discovery, (3) treatment strategy optimization, and (4) foundation models and LLMs (Figure 1). Through these AI‐driven strategies, we highlight how precision cancer immunotherapy can achieve improved patient outcomes. Finally, we critically discuss current limitations, including data heterogeneity, model interpretability, and barriers to clinical translation, providing actionable roadmaps to accelerate the integration of AI/ML into clinical practice.

## 2 AI for Patient Stratification in Cancer Immunotherapy

**FIGURE 2:**
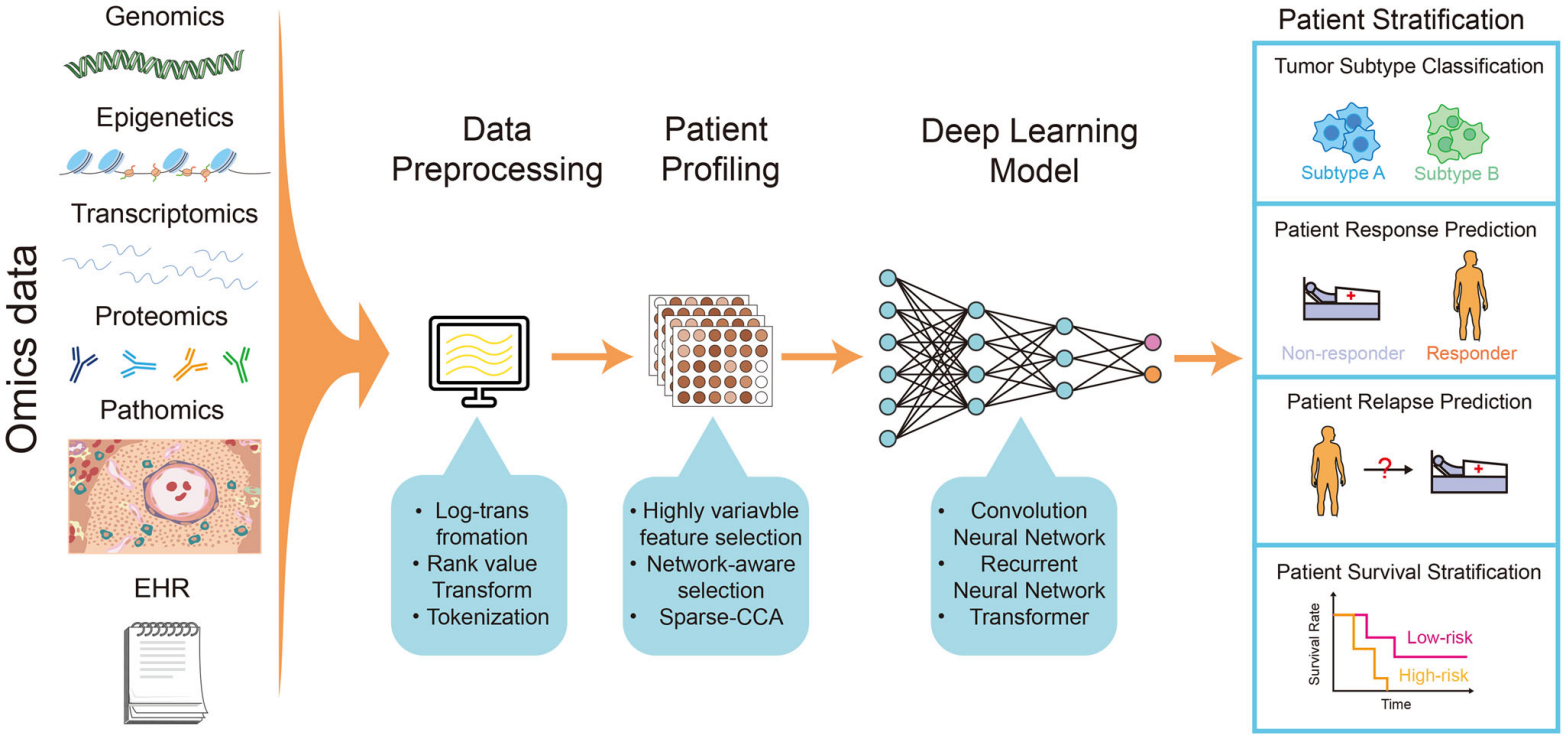
A framework of leveraging AI for patient stratification in cancer immunotherapy. Omics data are aggregated for rigorous processing to generate informative patient profiling. Following that, DL models leverage the patient profiling after feature selection to predict patient stratification. The preprocessing usually included log‐transformation, rank value transformation, and tokenization, etc. To select informative features, highly variable feature selection, network‐aware selection, and sparse‐CCA are implemented. Data are fed into an AI model, including Convolution Neural Network (CNN), Recurrent Neural Network (RNN), and Transformer, which are trained to generate patient‐level class assignments enabling tumor subtype classification, patient response prediction, patient relapse prediction, and prognostic risk grouping. Abbreviation: EHR, electronic health records; Sparse‐CCA, sparse canonical correlation analysis.

Patient stratification in cancer immunotherapy refers to the process of categorizing patients into distinct subgroups based on their tumor subtypes, likelihood of response, relapse, and survival rate to specific immunotherapy treatments. This stratification enables clinicians to match individual patients with the most appropriate therapeutic interventions, optimizing treatment efficacy while minimizing unnecessary exposure to ineffective or potentially harmful regimens. Given the established heterogeneity in immunotherapy responses, patient stratification has emerged as a critical component of AI/ML for cancer immunotherapy [44]. AI/ML computational approaches can be used to address a fundamental need in cancer immunotherapy, i.e., identifying which patients will benefit from specific immunotherapy regimens before treatment initiation, and monitoring their trajectory throughout the treatment course [45]. For instance, models could timely report potential immune‐related adverse events (irAEs) associated with immunotherapy treatment [46]. These AI/ML‐driven stratification methods can be broadly categorized into four paradigms: (1) cancer subtype classification, which categorizes tumors based on molecular and morphological features, (2) patient response prediction, which forecasts treatment outcomes by integrative baseline patient characteristics, tumor profiles, and immune signatures, (3) patient relapse prediction, which predicts the patient relapse rate for in time monitoring, and (4) patient survival stratification, which identifies risk groups to guide intensity clinical decisions. As illustrated in Figure 2, the computational workflow begins with aggregation of omics‐based data (e.g., genomics, epigenetics, transcriptomics, proteomics, pathomics, and EHR) at the patient level. These heterogeneous data undergo rigorous preprocessing, including log‐transformation, rank value transformation for distributional normalization, and tokenization for language model compatibility. This is followed by feature selection strategies, such as highly variable feature selection [47], network‐aware selection [48], and sparse canonical correlation analysis (sparse‐CCA) [49] to identify the most informative features. The processed data are then fed into diverse AI architectures, including convolutional neural networks (CNNs) [50] for capturing patterns in imaging or sequential dependencies in genomic data, recurrent neural networks (RNNs) [51] for modeling temporal treatment trajectories, or transformers [52] for learning complex cross‐modality relationships. By transforming these complex and multi‐dimensional datasets into actionable clinical insights through this systematic workflow, AI/ML models can establish a new paradigm in precision immunotherapy for patient‐tailored treatment design.

### 2.1 Tumor Subtype Identification

Tumor subtyping refers to the classification of cancers into distinct molecular and phenotypic categories that go beyond traditional histological classification. In cancer immunotherapy, accurate tumor subtyping is essential, as it forms a cornerstone of precision immunotherapy [53], as tumors with similar histological features can harbor vastly different molecular and immunological profiles that dictate therapeutic response [54]. This molecular heterogeneity directly influences whether a tumor will respond to specific immunotherapy treatments, making accurate tumor subtyping critical for treatment selection and outcome prediction. Computational approaches for cancer subtyping in immunotherapy contexts [55] have evolved dramatically, outperforming traditional single biomarkers like tumor mutational burden (TMB) and programming death ligand 1 (PD‐L1) expression [56]. These AI/ML models leverage heterogeneous data generated in modern oncology, ranging from histopathology slides to multi‐omics profiles, to uncover latent patterns that elude conventional statistics [57]. Importantly, different modeling paradigms are advantageous under distinct data availability, clinical constraints, and immunotherapy contexts. Accordingly, existing AI approaches for immunotherapy‐relevant tumor subtyping can be synthesized into four complementary paradigms: (1) *imaging‐based* that extracts morphological features from histopathology, (2) *omics‐based* that extracts molecular features from multi‐omics data, (3) *transformer‐based* that synthesizes heterogeneous molecular profiles, and (4) *federated learning* frameworks that enable privacy‐preserving collaboration across institutions. Each paradigm has characteristic strengths, limitations, and clinical niches.

*Imaging‐based* approaches for tumor subtyping have demonstrated remarkable success in extracting prognostic and predictive biomarkers directly from routine histopathology slides, offering a cost‐effective alternative to molecular testing for immunotherapy patient subgrouping. These approaches are particularly advantageous in immunotherapy settings where molecular profiling is time‐constrained, such as first‐line ICB decision‐making in routine clinical practice. Because digitized H&E slides are universally available, these models enable rapid, population‐scale patient stratification and can outperform traditional biomarkers in predicting immunogenic phenotypes such as microsatellite instability or immune‐inflamed tumor states. However, their reliance on weakly supervised labels and indirect biological proxies limits their suitability for mechanistic discovery or therapy engineering tasks, such as CAR‐T target selection. Chen et al. [58] developed a convolutional neural network (CNN)‐based pipeline to infer epithelial‐mesenchymal transition (EMT) status in clear‐cell renal cell carcinoma (ccRCC) by text‐mining 63 EMT‐related genes and clustering The Cancer Genome Atlas (TCGA) expression profiles for robust ground truth. Their tile‐level classifier achieved an AUC of 0.99 and translated to 74.9% accuracy at the sample level, offering a rapid, low‐cost readout to inform prognosis and immunotherapy decisions. Similarly, Kather et al. [59] showed that a compact ResNet could predict microsatellite instability (MSI) directly from routine H&E slides of colorectal and gastric cancers. Being trained on over 250 000 TCGA tiles, the model reached patient‐level AUCs of 0.77–0.84 and highlighted lymphocyte‐rich but poorly differentiated regions as morphologic hallmarks of MSI. One of the key advantages of image‐based approaches was the widespread availability of the required input data, namely the digitized slides, which are virtually available in all pathology workflows. However, a typical limitation of this type of approach was a lack of interpretability. The learned morphological features often represented complex, high‐dimensional patterns that are hard straightforward biological explanation. Zhao et al. [60] proposed PKMT‐Net, integrating pathological domain knowledge into a multi‐scale transformer architecture for lung cancer subtype prediction from pathological images. Rather than relying on data‐driven learning alone, PKMT‐Net explicitly incorporated high‐level medical knowledge into the model's architectural design, which significantly improved subtype prediction accuracy (0.996) compared to standard CNNs. However, pathological knowledge required deep manual annotations by domain experts, making the approach difficult to transfer across cancer types with different diagnostic criteria. Collectively, imaging‐based models are best suited for ICB response stratification and prognosis but remain less informative for cell‐engineering‐based immunotherapies, where direct molecular and cellular resolution is required.

*Omics‐based* approaches employ a multi‐stage DL architecture that explicitly separates feature extraction, dimensionality reduction, and prediction tasks. While imaging‐based models excel at scalability, omics‐based approaches are indispensable when immunotherapy decisions depend on precise molecular mechanisms, such as antigen presentation, immune evasion, or T‐cell dysfunction. This is particularly relevant for CAR‐T, TCR‐T, and therapeutic vaccine development, where pathway‐level and cell‐state resolution directly inform target selection and therapy design. Building on the progress achieved with a single‐omics AI framework, it has become clear that mono‐modality data are inherently limited in capturing the multi‐dimensional interactions governing tumor‐immune dynamics [61]. Single‐omics approaches often overlook critical regulatory layers, including epigenetic modifications, microbiome influences, and spatial contexts. It fails to reconcile the profound heterogeneity present across cellular and tissue levels. Multi‐omics integration addresses these limitations by unifying diverse data modalities into a systems‐level framework [62]. These modalities encompass bulk and single‐cell transcriptomics, somatic genomics, DNA methylation and chromatin accessibility profiles, radiologic and histopathologic imaging, microbiome composition, and spatially resolved expression maps. Advanced AI and ML algorithms provide the computational infrastructure to harmonize these heterogeneous inputs, extract latent cross‐modal features, and decode complex biological relationships [63]. The hybrid frameworks combine statistical methods for cross‐omics integration with a deep neural network (DNN) for pattern recognition. Zhao et al. [64] developed a two‐stage CNN pipeline for triple‐negative breast cancer (TNBC) that first segmented histologic components and then fed tissue‐specific image tiles into a second CNN to predict underlying transcriptomic read‐outs. This approach discriminated against four TNBC molecular subtypes, including basal‐like immune‐suppressed, immunomodulatory, luminal‐androgen‐receptor, and mesenchymal‐like, achieving AUC values ranging from 0.73 to 0.93. An auxiliary survival network that fused learned image features with key clinical covariates further stratified patients into prognostically distinct groups, and a real‐time PD‐L1 screening module achieved an AUC of 0.74, demonstrating the pipeline's potential for rapid subtype assignment and treatment guidance. Huang et al. [65] introduced DSCCN (differential sparse canonical‐correlation‐analysis network), a three‐step framework that identified reliable breast cancer subtypes from high‐dimensional multi‐omics data. They firstly filtered differential expression genes and DNA methylomes from TCGA cohorts and then implemented sparse CCA to identify pairs of DE features whose expression or methylation covaries across omics, capturing cross‐layer biology that single‐omics models overlook. Finally, they trained a multi‐task attention‐guided deep neural network across each omics, which outperformed concatenation, ensemble, and knowledge‐driven baselines, confirming that explicitly modeling inter‐omics correlations boosted subtype stratification accuracy. This method explicitly incorporated statistical constraints (e.g., sparse CCA) or domain‐specific preprocessing (histology segmentation) to reduce the data hunger of DL and improve convergence on limited training sets. While these approaches relied on predefined feature extraction strategies, which could miss emergent patterns and prevent full leveraging of task‐specific signals to guide representational learning. Compared with purely image‐based approaches, omics‐driven subtyping models demonstrate stronger mechanistic interpretability and transferability across immunotherapy modalities. However, they are more sensitive to batch effects, missing modalities, and limited cohort sizes, necessitating careful preprocessing and regularization to avoid overfitting.

Another group of approaches is *transformer‐based* methods, which leverage attention mechanisms to synthesize complementary molecular data types to capture biological complexity beyond what single data modalities can reveal. These models represent a high‐capacity, high‐risk paradigm, designed to capture global, non‐linear dependencies across genes or omics layers without predefined feature engineering. Their principal advantage lies in unbiased representation learning and the potential discovery of emergent subtype‐defining programs that are invisible to linear or modular models. For transformer‐based multi‐omics integration, the core operation can be summarized by scaled dot‐product attention, where the choice of tokenization (e.g., genes, modality‐specific features, or patient‐level patches) directly determines which pairwise dependencies are representable and thus which biological interactions can be expressed in the learned embedding. This flexibility is most beneficial in large, heterogeneous cohorts where cross‐modality interactions are a dominant source of predictive signal. For graph‐based formulations, a representative message‐passing update makes explicit that performance gains arise not from “nonlinearity” per se but from neighborhood aggregation under a task‐relevant adjacency $\tilde{A}$. These adjacencies include patient similarity graphs per omics layer, cell‐cell communication topology, or spatial neighborhoods, enhancing robustness over feature‐only baselines when labels are sparse but relational structure carries a stable biological signal. For the self‐supervised contrastive learning frameworks, it can be grounded by defining positive/negative pair construction, serving as an encoding of clinically meaningful invariances (batch effects, modality dropout, technical noise). Importantly, these assumptions were treated as testable because invariant‐preserving augmentations may inadvertently align phenotypically distinct subtypes with convergent molecular profiles, propagating erroneous similarity and compromising interpretability in downstream biomarker discovery. Specifically, Khan and Lee developed DeepGene (2023) [66], a method using multi‐head self‐attention mechanisms to gene expression data for pan‐cancer subtype classification, achieving superior performance over traditional machine learning and state‐of‐the‐art computational methods. DeepGene tokenized gene expression levels and delineated pairwise relationships between all genes through a self‐attention mechanism. It learned subtype‐defining gene signatures through an end‐to‐end learning framework without requiring domain knowledge of known cancer pathways, enabling unbiased biomarker discovery. However, DeepGene required training a massive number of parameters in the model, bringing severe overfitting risks when training cohorts were small, particularly for rare cancer subtypes with limited samples. Expanding beyond single‐omics analysis, Wang et al. [67] proposed MOSEGCN, combining transformer multi‐head self‐attention mechanisms with graph convolutional networks (GCN) for integrating mRNA, microRNA, and DNA methylation data for breast cancer subtyping. The model's innovation lay in constructing patient similarity graphs from each omics type separately, then using GCN layers to aggregate information from similar patients, while transformer attention layers to learn cross‐omics relationships. Pan et al. [68] introduced DEDUCE, an unsupervised multi‐omics subtyping framework that couples a symmetric multi‐head attention encoder (SMAE) with a subtype‐decoupled contrastive clustering objective. Simultaneously computing sample similarities in both feature and embedding spaces, it made clustering robust under the noise, sparsity, and scale mismatch typical of cancer multi‐omics profiles. Concretely, DEDUCE first standardized and concatenated multi‐omics features, then applied position encoding and a transformer‐style multi‐head self‐attention encoder, encouraging cross‐omics feature sharing to extract contextual, long‐range dependencies while suppressing irrelevant signals. Rather than performing “embed‐then‐cluster” as a disconnected two‐stage pipeline, DEDUCE generated two augmented views of each sample, projected them with an MLP into normalized embedding spaces, and optimized a cosine‐similarity cross‐entropy objective that treated same‐sample views as positives and other samples as negatives. A key methodological refinement was its decoupled contrastive formulation, by removing positive pairs from the denominator of the InfoNCE‐style loss. DEDUCE aimed to mitigate the “negative‐positive coupling” effect that can otherwise over‐emphasize negatives and weaken gradients for true positives, thereby improving cluster tightness for putative subtypes. DEDUCE outperformed ten benchmarking DL models on simulated, single‐cell, and bulk cancer multi‐omics benchmarks, uncovering six robust acute myeloid leukemia (AML) subtypes with enhanced interpretability of subtype‐defining molecular signatures. The self‐supervised learning framework of MOSEGCN leveraged both labeled and unlabeled samples through a consistency regularization loss that encouraged similar patients to receive similar predictions, effectively utilizing abundant unlabeled data to improve model robustness and generalizability. But this self‐supervised learning assumption might not stand when phenotypically distinct subtypes share molecular similarities, leading to the propagation of incorrect labels. Across simulated, single‐cell, and TCGA multi‐omics benchmarks, the authors report improved clustering quality over multiple deep clustering baselines and use downstream differential analysis and enrichment to interpret the discovered clusters, including the identification of six AML subtypes. Despite their strong benchmark performance, transformer‐based subtyping models remain vulnerable to overfitting in rare cancer subtypes and may propagate spurious subtype assignments when phenotypically distinct tumors share convergent molecular signatures, limiting their reliability in low‐sample or highly imbalanced immunotherapy cohorts.

The fourth type of approaches for tumor subtyping in cancer immunotherapy is *federated learning* methods, which represent an emerging paradigm that addresses critical data‐sharing barriers in multi‐institutional cancer research while maintaining patient privacy. Federated learning is uniquely suited for large‐scale, cross‐institutional immunotherapy studies, where privacy regulations prohibit centralized data aggregation, such as multinational ICB biomarker discovery or rare tumor subtype analysis. However, its performance degrades when participating sites exhibit severe class imbalance or inconsistent annotation standards, which are frequently encountered in real‐world immunotherapy datasets. Cai et al. [69] introduced ProCanFDL, a federated deep‐learning framework that aggregated local model updates from 30 proteomic cohorts (7525 biospecimens) to classify 14 histopathologic subtypes under strict privacy constraints. The global classifier improved accuracy by 43% over site‐specific models and matched fully centralized training on a 625‐sample hold‐out set, demonstrating the feasibility of privacy‐preserving, large‐scale proteomic AI collaborations. This federated approach offered substantial advantages in dataset scaling and diversity without compromise of data privacy. However, federated learning introduced technical complexity in model synchronization, required substantial computational infrastructure at each participating site, and might underperform when different local datasets exhibited imbalanced class distributions.

Detailed comparisons of these four categories of approaches for tumor subtyping in cancer immunotherapy are summarized in Table 1. Imaging‐based deep learning models offer unparalleled scalability and clinical accessibility, making them well‐suited for large‐scale immunotherapy stratification when molecular data are unavailable. However, their reliance on weak supervision and indirect morphological proxies limits mechanistic interpretability and robustness to domain shift. Omics‐based deep learning models provide higher biological resolution by directly modeling molecular layers and their interactions, enabling more mechanistically grounded tumor subtyping, but their performance is constrained by data quality, batch effects, and limited cohort sizes. Transformer‐based models further expand representational capacity through attention‐driven, end‐to‐end learning of global nonlinear dependencies, facilitating discovery of emergent immunotherapy‐relevant patterns, yet their high parameterization increases overfitting risk in small or imbalanced datasets and complicates biological interpretation. Federated learning frameworks address privacy and data‐sharing barriers by enabling multi‐institutional collaboration and improved cohort diversity, particularly for rare cancers, but introduce substantial system complexity and may underperform under heterogeneous data distributions or severe class imbalance. Collectively, these paradigms embody distinct trade‐offs between scalability, interpretability, biological fidelity, and generalizability, underscoring the need for context‐dependent model selection rather than a one‐size‐fits‐all solution.

**TABLE 1:** A summary of AI models developed for tumor subtype classification in cancer immunotherapy. Models are listed chronologically within each model category. The Key Features and Limitations column presents the advantages and disadvantages of each approach category in “A; B” format, where A represents the advantages and B represents the disadvantages.

Model type	Method	Key idea	Year	References	Key features and limitations
Imaging‐based DL	ResNet‐MSI	ResNet	2019	Kather et al. [59]	Widely available, cost‐effective; Lack of interpretability
	EMT‐CNN	CNN	2021	Chen et al. [58]	
	PKMT‐Net	Vision transformer	2025	Zhao et al. [60]	
Omics‐based DL	Two‐stage CNN	CNN	2024	Zhao et al. [64]	Improved convergence; Reliant on predefined feature
	DSCCN	DNN	2024	Huang et al. [65]	
Transformer‐based	DeepGene transformer	Transformer	2023	Khan and Lee [66]	Without requiring domain knowledge; Prone to overfit with small cohorts
	MOSEGCN	GCN	2024	Wang et al. [67]	
	DEDUCE	Transformer encoder	2024	Pan et al. [68]	
Federated learning	ProCanFDL	Federated learning	2025	Cai et al. [69]	Data privacy preserved; Technically complicated

Abbreviations: DL, deep learning; EMT, epithelial‐mesenchymal transition; CNN, convolutional neural network; MSI, microsatellite instability; PKMT‐Net, pathological knowledge‐integrated multi‐scale transformer network; DSCCN, differential sparse canonical‐correlation‐analysis network; ccRCC, clear‐cell renal cell carcinoma; CRC, colorectal cancer; TNBC, triple‐negative breast cancer; AML, acute myeloid leukemia; ICI, immune checkpoint inhibitor; ICB, immune checkpoint blockade; H&E WSI, hematoxylin and eosin whole slide image; AUC, area under the receiver operating characteristic curve.

### 2.2 Patient Response Prediction

Predicting patient response refers to the prospective assessment of whether an individual patient will achieve benefit from a specific immunotherapy regimen, encompassing complete response, partial response, stable disease, or progressive disease according to standard criteria [70]. This prediction is critical in cancer immunotherapy because response rates among eligible individuals vary dramatically depending on tumor type and treatment regimen. Early and accurate identification of responders can optimize clinical outcomes by enabling personalized treatment strategies, minimizing exposure to ineffective therapies and associated toxicities, and reducing healthcare costs [71]. However, immunotherapy responses are governed by multiscale interactions within the TME, host genetics, and systemic immune factors, which exhibit considerable inter‐ and intra‐patient variability [72]. In addition, immunotherapies can cause paradoxical phenomena like pseudo‐progression, for which conventional response criteria developed for chemotherapy or other therapies may misclassify these cases [73]. To address these challenges, AI‐driven approaches for immunotherapy response have been developed, which could be categorized into four types: (1) *regression‐based* models prioritizes interpretability and clinical practicality through feature engineering, (2) *ensemble ML* approaches that combine multiple classical algorithms for robust predictions through consensus integration, (3) *prior knowledge‐based* AI models that embed biological domain expertise into neural architectures, and (4) *foundation models* that leverage large‐scale pre‐training to capture universal patterns across cancer types. We explicitly discuss when each approach is preferred, its typical failure modes, and their relevance to specific immunotherapy modalities.

*Regression‐based* models for immunotherapy response prediction subtyping prioritize simplicity and transparency by engineering a small set of interpretable features from multimodal data, enabling rapid implementation and real‐time clinical decision support. This is critical in tasks such as first‐line ICB decision‐making in routine oncology practice. Their reliance on a small number of well‐characterized features allows clinicians to directly interrogate model logic and facilitates prospective validation in multi‐center trials. For example, Chang et al. [74] carried out a multi‐institutional evaluation of twenty ML pipelines across 2,881 ICB‐treated and 841 non‐ICB patients spanning eighteen tumor types, ultimately defining the LORIS (Logistic Regression‐based Immunotherapy‐response Score). LORIS integrated six routinely available features, namely TMB, human leukocyte antigen (HLA)‐class‐I evolutionary divergence, copy‐number‐alteration fraction, neutrophil‐to‐lymphocyte ratio, serum albumin, and sex, to predict immunotherapy benefit with high accuracy even in tumors exhibiting low PD‐L1 expression or low TMB. Its rapid calculability enabled dynamic monitoring: a declining LORIS score during treatment could trigger timely therapy adjustments. Compared with single biomarkers such as PD‐L1 expression or TMB alone, regression‐based scores like LORIS consistently demonstrate improved discrimination by integrating orthogonal immune, genomic, and systemic features. However, their performance plateaus when response determinants reside in high‐dimensional molecular or spatial patterns that are not captured by handcrafted variables.

*Ensemble ML* approaches rely on combining multiple classical ML algorithms to achieve robust predictions through consensus or weighted integration. These approaches prioritize algorithmic diversity and transparent feature selection, making them particularly suitable for moderate‐sized datasets and clinical contexts where model interpretability is paramount, and no single modeling assumption dominates. Liu et al. [75] catalogued 131 immunogenic‐cell‐death (ICD) genes in ccRCC by synthesizing single‐cell and bulk transcriptomes, genomic alterations, and clinical metadata, then applied an ensemble of ten machine‐learning algorithms (including random forest, elastic net, and CoxBoost) to derive an ICD‐related signature (ICDRS). ICDRS robustly stratified overall, progression‐free, and disease‐specific survival across internal and external cohorts, outperforming conventional clinicopathologic models, and multi‐omics analyses revealed that high‐ and low‐ICDRS tumors diverged in TMB, pathway activation, and immune‐cell infiltration. Notably, high‐risk ICDRS cases exhibited elevated immunophenotypic scores and were predicted to derive greater benefit from ICB. Similarly, extending to melanoma, Zhao et al. [76] integrated five omics layers (mRNA, lncRNA, miRNA, DNA methylation, and somatic mutations), clinical annotations, and single‐cell RNA‐seq profiles within a comprehensive ML workflow encompassing 101 algorithmic combinations. Their resulting machine‐learning‐driven signature (MLDS) delineated three molecular melanoma subtypes with a concordance index exceeding 0.80, pinpointed AGPAT2 as an immunologically actionable target, and showed that low‐MLDS tumors harbored enriched immune infiltration and were predicted to respond more favorably to PD‐1 or CTLA‐4 blockade. The modular nature of ensemble approaches facilitated integration of domain knowledge and adaptation to different cancer types without architecture redesign. Despite their strong empirical performance, ensemble models typically rely on late‐stage fusion of independently processed modalities, limiting their ability to capture higher‐order cross‐omics interactions. As a result, they are less suitable for mechanistic inference or for immunotherapy modalities such as CAR‐T or cancer vaccines, where coordinated molecular programs rather than aggregate risk scores often drive therapeutic efficacy.

*Prior knowledge‐based* AI models integrate established domain knowledge, such as pathway networks, gene interactions, or tissue compartment structures, into DL architectures to constrain the hypothesis space, enhancing both interpretability and clinical prediction confidence. This paradigm is particularly valuable when the goal extends beyond prediction to mechanistic insight or therapeutic optimization. Specifically, Jiang et al. [77] introduced a “biology‐guided” deep‐learning (BgDL) model that constrained intermediate network representations to correspond to TME compartments, including immune‐cell infiltration, producing interpretable links to recognize radiologic patterns. In an international, multi‐center gastric cancer cohort, BgDL outperformed PD‐L1 immunohistochemistry and mismatch‐repair status and identified a subset of mismatch‐repair‐deficient tumors that failed to respond to single‐agent PD‐1 blockade. Zhao et al. [78] leveraged a graph‐based deep‐learning framework (ICInet) anchored in curated gene‐gene interaction and pathway networks to forecast PD‐1/PD‐L1 blockade outcomes from bulk RNA‐seq across more than 600 patients with melanoma, gastric, and bladder cancers. By embedding biological knowledge directly into model architecture, ICInet achieved an average AUC of 0.85, exceeding conventional biomarkers, and pinpointed regulatory circuits and cell‐state programs underlying response heterogeneity. On one hand, these biology‐guided approaches generated biologically meaningful hypotheses about resistance mechanisms and therapeutic vulnerabilities that could guide immunotherapy strategies. On the other hand, biology‐guided models are constrained by the completeness and correctness of existing knowledge bases, which may underrepresent tissue‐specific, spatially organized, or treatment‐induced immune programs. Consequently, these approaches may underperform in emerging immunotherapy contexts where resistance mechanisms are poorly characterized or rapidly evolving.

*Foundation models* leverage massive datasets across diverse cancer types to learn universal representations that generalize across tumor types. The pre‐trained representations can encode shared immunological structure across cancer types, treatment regimens, and clinical settings. Specifically, Shen et al. [79] developed COMPASS, which was a concept‐bottleneck foundation model motivated by a persistent limitation that current ICI biomarkers, such as PD‐L1, TMB, or fixed transcriptomic scores, often fail to transfer across cancer types. COMPASS addressed this by interposing a biologically grounded intermediate representation between transcriptomes and response prediction, which was a transformer‐based gene encoder that maps bulk RNA‐seq profiles into context‐aware gene representations. The representations were then passed through a hierarchical concept projector that aggregates genes into granular immune‐relevant concepts and compresses them into 44 high‐level TIME concepts spanning immune cell states, TME interactions, and pathways. At deployment, COMPASS used partial fine‐tuning that adapts only a small subset of layers to small ICI cohorts, mitigating overfitting relative to end‐to‐end deep models while preserving the pre‐trained TIME structure. Trained on data from over thirty‐three types of cancer, COMPASS outperformed twenty‐two established biomarkers and ML signatures across seven malignancies and six ICI cohorts, achieving a hazard ratio of approximately 4.7 in distinguishing responders from non‐responders. Its concept layer further generated patient‐specific “response maps” that revealed distinct resistance circuits amenable to therapeutic targeting. Spatial‐resolved foundation models capture critical TME context and cellular neighborhood dynamics that bulk analyses missed, revealing spatial biomarkers of response. Despite their strong cross‐cohort generalization and conceptual interpretability, foundation models currently face substantial translational barriers, including the need for massive, harmonized training datasets, standardized preprocessing pipelines, and substantial computational resources. These requirements limit near‐term clinical deployment but position foundation models as long‐term infrastructure for population‐scale immunotherapy modeling.

Detailed comparisons of these four categories of approaches for patient response prediction in cancer immunotherapy are summarized in Table 2. The *regression‐based* models are clinically transparent and easy to deploy, due to their interpretable coefficients and routinely available features. However, the performance depends heavily on manual feature engineering and prior biomarker knowledge, limiting the discovery of novel predictions. *Ensemble learning* is robust to noise and cohort heterogeneity, while ensembles typically rely on late fusion and shallow interactions, limiting their ability to capture high‐order, cross‐omics mechanistic relationships. *Prior knowledge‐based* approaches are mechanistically interpretable and biologically grounded, improving their convergence on limited data and yielding clinically meaningful explanations. However, reliance on existing knowledge bases introduces bias and limits the discovery of novel mechanisms. *Foundation models* have strong advantages in generalization and transferability, enabling consistent immunotherapy response prediction across tumor types and cohorts. At present, their main limitations are practical rather than conceptual. The development of foundation models requires massive, harmonized datasets and extensive computational resources.

**TABLE 2:** A summary of AI models developed for immunotherapy patient response prediction. Models are listed chronologically within model categories. The Key Features and Limitations column presents the advantages and disadvantages of each approach category in “A; B” format, where A represents the advantages and B represents the disadvantages.

Model type	Method	Key ideas	Year	References	Key features and limitations
Regression‐based	LORIS	Logistic regression	2024	Chang et al. [74]	Simple and transparent; Rely on manual feature engineering
Ensemble learning	ICDRS	RF, Elastic net, and CoxBoost	2023	Liu et al. [75]	Highly robust; Struggle to capture high‐order interactions
	MLDS	Ensemble learning of 11 ML algorithms	2025	Zhao et al. [76]	
Prior knowledge‐based	BgDL	CNN	2023	Jiang et al. [77]	Integrate established domain knowledge; Inherit limitations and biases.
	ICInet	GNN	2023	Zhao et al. [78]	
Foundation model	COMPASS	Transformer	2025	Shen et al. [79]	Excel at generalization; Requires massive datasets.

Abbreviations: LORIS, Logistic Regression‐based Immunotherapy‐response Score; ICDRS, immunogenic cell death‐related signature; MLDS, machine learning‐driven signature; BgDL, biology‐guided deep learning; COMPASS, comprehensive patient‐centric analysis; RF, random forest; ML, machine learning; CNN, convolutional neural networks; GNN, graph neural networks; ccRCC, clear‐cell renal cell carcinoma; GC, gastric cancer; ICI, immune checkpoint inhibitor; ICB, immune checkpoint blockade; TME, tumor microenvironment; AUC, area under ROC curve; scRNA‐seq, single‐cell RNA‐sequencing; CT, computed tomography; DNA‐meth, DNA methylation.

### 2.3 Patient Relapse Prediction

Patient relapse prediction in cancer immunotherapy refers to the prospective identification at high risk of disease recurrence after initial treatment response or surgical resection, and the temporal forecasting of when relapse is likely to occur. Because relapse is intrinsically a time‐to‐event problem, robust modeling must explicitly accommodate censoring, heterogeneous follow‐up intensity, and clinically actionable lead‐time, rather than treating relapse as a static endpoint. Early detection of relapse following therapy is critical for timely intervention, yet current post‐operative surveillance strategies are largely “one‐size‐fits‐all”. AI frameworks that incorporate multi‐layer of omics data are uniquely suited to this problem because they can learn non‐linear risk trajectories that traditional Cox proportional hazards or logistic regression models struggle to represent. Jung et al. [80] built RADAR CARE, a practical, transformer‐based deep‐learning model that continuously predicts 1‐year recurrence using multimodal, real‐world data for early‐stage NSCLC patients. It continuously predicted 1‐year recurrence using baseline clinicopathologic variables and time‐updated laboratory and radiology‐text features. Researchers retrospectively enrolled 14 177 stage I–III patients (10,262 I; 2,380 II; 1,703 III) treated between 2008 and 2022, integrating 64 baseline clinical, pathologic, and molecular variables with longitudinal laboratory results and radiologic interpretations collected during follow‐up. The model outputted a RADAR score in real time; baseline means were 0.324 for stage I, 0.660 for stage II, and 0.824 for stage III. Across all stages, the AUC for predicting relapse within the subsequent year was 0.854, with 86.0% sensitivity and 71.3% specificity (AUC 0.872 in stage I, 0.737 in stage II, 0.724 in stage III). Four distinct RADAR score trajectories revealed that personalized surveillance schedules and early escalation to immunotherapy could be guided by rising risk. By integrating baseline molecular features (genomics alterations and pathological subtypes) with dynamic laboratory trends (tumor markers, inflammatory indices) and serial imaging assessments, RADAR captured both intrinsic tumor biology and evolving host‐tumor interactions that jointly determined relapse risk. In melanoma treated with neoadjuvant drugs, Chan et al. [81] showed that post‐surgery ctDNA positivity was predictive of recurrence (specificity 100%; sensitivity 44%), while ctDNA zero‐conversion identified patients with extremely low relapse risk, directly motivating ctDNA‐guided escalation/de‐escalation strategies after immunotherapy. Complementary multimodal frameworks further improve risk stratification when ctDNA alone is limited by low shedding. Tran et al. [82] integrated serial ctDNA clearance with CT‐derived 3D tumor volume in resected early‐stage NSCLC, where ctDNA detected after standard‐of‐care therapy was associated with substantially worse outcomes (RFS HR 2.80; OS HR 3.99), and the combined ctDNA and volume model separated high‐ versus low‐risk groups with external validation, which was an explicit improvement over single‐modality monitoring for guiding intensified follow‐up and adjuvant systemic therapy. Finally, relapse prediction becomes especially decision‐critical in treatment discontinuation settings, where the endpoint is relapse after stopping ICI rather than initial response. Noh et al. [83] addressed this in advanced melanoma by combining clinical variables with explainable AI‐derived digital pathology features from pre‐treatment H&E whole‐slide images and gene‐expression markers, achieving 84.6% predictive accuracy with a multivariate adaptive regression spline model. Taken together, transformer‐style longitudinal fusion models are emerging as relapse‐prediction paradigms in immunotherapy, and they naturally align with relapse‐focused evaluation choices such as surveillance and escalation actions.

### 2.4 Patient Survival Stratification

Patient survival stratification in cancer immunotherapy aims to categorize patients into distinct risk groups according to their predicted survival outcomes following immunotherapy treatment [84]. This stratification goes beyond binary response prediction to estimate the duration and probability of overall survival, progression‐free survival, or disease‐specific survival, enabling risk‐adapted treatment decisions. Accurate stratification enables clinicians to tailor therapeutic regimens to individual risk profiles, thereby maximizing efficacy, minimizing irAE, and improving overall survival and quality of life [46]. However, traditional stratification approaches, which rely on single biomarkers or low‐dimensional statistical models, are insufficient to capture these complex hidden patterns [44]. Recently, a series of AI‐driven stratification frameworks [85, 86, 87, 88] have been developed, which can be categorized into three progressively sophisticated paradigms based on their architectural complexity and data integration strategies: (1) *ensemble learning* approaches that combine traditional classifiers to identify robust cell‐type‐specific signatures, (2) *attention‐based DL* models, and (3) *spatial‐involved* methods.

*Ensemble learning* approaches leverage the complementary strengths of multiple traditional algorithms, such as random forests, support vector machine (SVM), and gradient boosting, to identify robust, cell‐type‐specific molecular signatures for patient survival stratification. Liu et al. [85] integrated ∼430,000 single cells with nine bulk RNA‐seq immunotherapy cohorts and ensembled eight ML algorithms to derive M.Sig, a macrophage‐centered signature capturing an APOE^+^ resistance program. M.Sig demonstrated consistent predictive performance across tumor types, offering a candidate companion diagnostic and nominating APOE blockade as a strategy to sensitize refractory tumors. Similarly, Zhou et al. [86] combined bulk and single‐cell RNA‐seq in neuroblastoma to identify cell‐cycle‐driven “hub” genes enriched in a fast‐cycling, poorly differentiated malignant subcluster that fostered an immunosuppressive microenvironment. Through multiple ML pipelines, they distilled a Malignant Subcluster‐Related Signature (MSRS) that outperformed conventional clinical factors and existing gene panels in risk stratification. By coupling single‐cell defined tumor subsets with ML‐derived gene signatures, MSRS intrinsically encoded both tumor‐intrinsic proliferation programs and immune‐context features. These approaches offered substantial advantages in robustness and generalizability, as consensus predictions across diverse algorithms to reduce vulnerability to individual model bias and overfitting. However, the ensemble methods could only capture linear or simple non‐linear relationships that might miss higher‐order interactions across omics data.

Advancing to more complex models, *attention‐based DL* models employ self‐attention mechanisms to automatically learn hierarchical representations of biological programs, capturing complex dependencies among genes and pathways. Tang et al. [87] constructed a pyroptosis‐related (PR) risk system for HCC by integrating single‐cell and bulk RNA‐seq data. They first curated 105 pyroptosis‐related genes and computed a per‐cell pyroptosis activity score and a per‐patient pyroptosis activity score. After that, they defined “candidate hub” genes as those differentially expressed between high‐ vs. low‐pyroptosis strata at both cell‐level and patient‐level, thereby prioritizing features that are robust to cell‐mixture effects. They next applied two complementary feature‐selection schemes (SVM‐RFE and LASSO) and intersected their outputs, yielding a minimal three‐gene signature (ERGIC3, STAT4, MGMT). They used the following formula to convert the expression into a continuous PR risk score via a Cox‐derived linear form: $\mathrm{RP}\_\mathrm{risk}=\sum_{i}\beta_{i}\cdot \;\mathrm{Exp}r_{i}$. This formula showed the transparent attribution of how each gene contributed to hazard while preserving the survival‐modeling semantics. Importantly, the “attention‐based DNN” in this study functioned less as a black‐box replacement for the Cox score. It served as an explicit verification that learns a patient‐specific reweighting decision boundary. In the immunotherapy‐relevant setting, they further linked PR risk to ICB responsiveness using TIDE and external ICI cohorts. This proved the claim that the score encoded clinically meaningful immune constraints rather than generic proliferation risk. But these approaches face challenges when the training cohorts are small, a frequent scenario for rare cancer subtypes with limited patient samples, creating severe overfitting risks.

*Spatial‐involved* methods focus on integrating cutting‐edge, highly multiplexed imaging techniques and spatial information with AI/ML to capture TME and cell‐cell interactions. For example, Blise et al. [88] demonstrated the power of highly multiplexed, spatially resolved proteomics by profiling more than 2.5 million single cells from 306 pancreatic ductal adenocarcinoma regions, half treatment‐naïve and half post‐neoadjuvant CD40 agonist therapy. Elastic net trained on a thousand quantitative TME features accurately classified treatment status and predicted disease‐free survival, with superior outcomes linked to neighborhoods enriched in CD44^+^ CD4^+^ Th1 cells exhibiting high proliferation, antigen experience, and cytotoxicity. These methods provide a transformative advantage by capturing spatial biomarkers, such as immune infiltration patterns, tumor‐immune interfaces, and cellular neighborhood compositions. However, these methods faced formidable translation barriers, as the current profiling technologies were expensive and required specialized equipment, unavailable to most clinical laboratories. The interpretation of spatial neighborhood patterns was another challenge, which needed clinical expertise.

Detailed comparisons of these three categories of approaches for patient survival stratification in cancer immunotherapy are summarized in Table 3. *Ensemble learning* models enhance robustness and stability by aggregating predictions from multiple classical machine‐learning algorithms, making them well‐suited for moderate‐sized and heterogeneous immunotherapy cohorts; however, their reliance on shallow or late‐stage feature fusion limits their ability to capture high‐order nonlinear interactions and coordinated biological programs. *Attention‐based DL* models introduce greater expressive power by learning nonlinear, context‐dependent relationships among genes or pathways, enabling the discovery of latent risk programs relevant to immunotherapy response, but this flexibility increases overfitting risk in small or imbalanced datasets and complicates clinical interpretability. *Spatially‐involved* models further improve biological realism by incorporating tissue architecture and cellular neighborhood information, capturing tumor–immune interactions that are invisible to spatially agnostic approaches; nevertheless, their dependence on specialized spatial data, platform‐specific preprocessing, and complex feature engineering presents substantial barriers to scalability, standardization, and routine clinical translation.

**TABLE 3:** A summary of AI models developed for patient survival stratification in cancer immunotherapy. Models are listed chronologically within model categories. The Key Features and Limitations column presents the advantages and disadvantages of each approach category in “A; B” format, where A represents the advantages and B represents the disadvantages.

Model type	Method	Key ideas	Year	References	Key features and limitations
Ensemble Learning	M.Sig	8 ML classifiers	2024	Liu et al. [85]	Highly robust; Miss high‐order interactions
	MSRS	10 ML algorithms	2025	Zhou et al. [86]	
Attention‐based	Pyroptosis Risk System	Multi‐head attention‐mechanism	2024	Tang et al. [87]	Excel at non‐linear models; Overfitting in small datasets
Spatial‐involved	Spatial‐IMC ML	Elastic net	2024	Blise et al. [88]	Integrate neighborhood information; Translation barriers

Abbreviations: DL, deep learning; M.Sig, macrophage signature; MSRS, Malignant Subcluster‐Related Signature; IMC, imaging mass cytometry; ML, machine learning; MHC‐I, major histocompatibility complex class I; PD‐L1, programmed death‐ligand 1; ICI, immune checkpoint inhibitor; HCC, hepatocellular carcinoma; OS, overall survival; DFS, disease‐free survival; scRNA‐seq, single‐cell RNA‐sequencing; APOE, apolipoprotein E; Th1, T helper 1.

## 3 AI for Biomarker Identification in Cancer Immunotherapy

Biomarkers in cancer immunotherapy are measurable biological characteristics, including genes, proteins, or other biological substances indicative of disease presence or progression, which represent molecular or cellular characteristics derived from the tumor, the host immune system, or their bidirectional interactions within the TME. These biomarkers vary by tumor types, immune microenvironments, or host factors, and serve as diagnostic, prognostic, or predictive indicators of responsiveness to cancer immunotherapy. Despite the transformative potential of immunotherapy in treating cancers such as melanoma [89], head and neck cancer [90], bladder [91, 92], kidney [93], and advanced NSCLC [94], clinical benefits remain limited to certain patient subsets. Presently, many patients receive immunotherapy without reliable biomarkers, resulting in unnecessary toxicity and limited therapeutic benefits [95]. Conventional biomarker identification has relied on PD‐L1 immunohistochemistry and TMB measurements, which assess immune activity and tumor immunogenicity [96, 97]. However, these assays are confounded by substantial tumor and immune microenvironment heterogeneity, inter‐assay variability, and limited predictive power, failing to capture the dynamic and spatially diverse tumor–immune interactions that underlie therapeutic response [98]. These shortcomings underscore the need for integrative, data‐driven biomarker discovery approaches to guide personalized immunotherapy and improve clinical outcomes.

**FIGURE 3:**
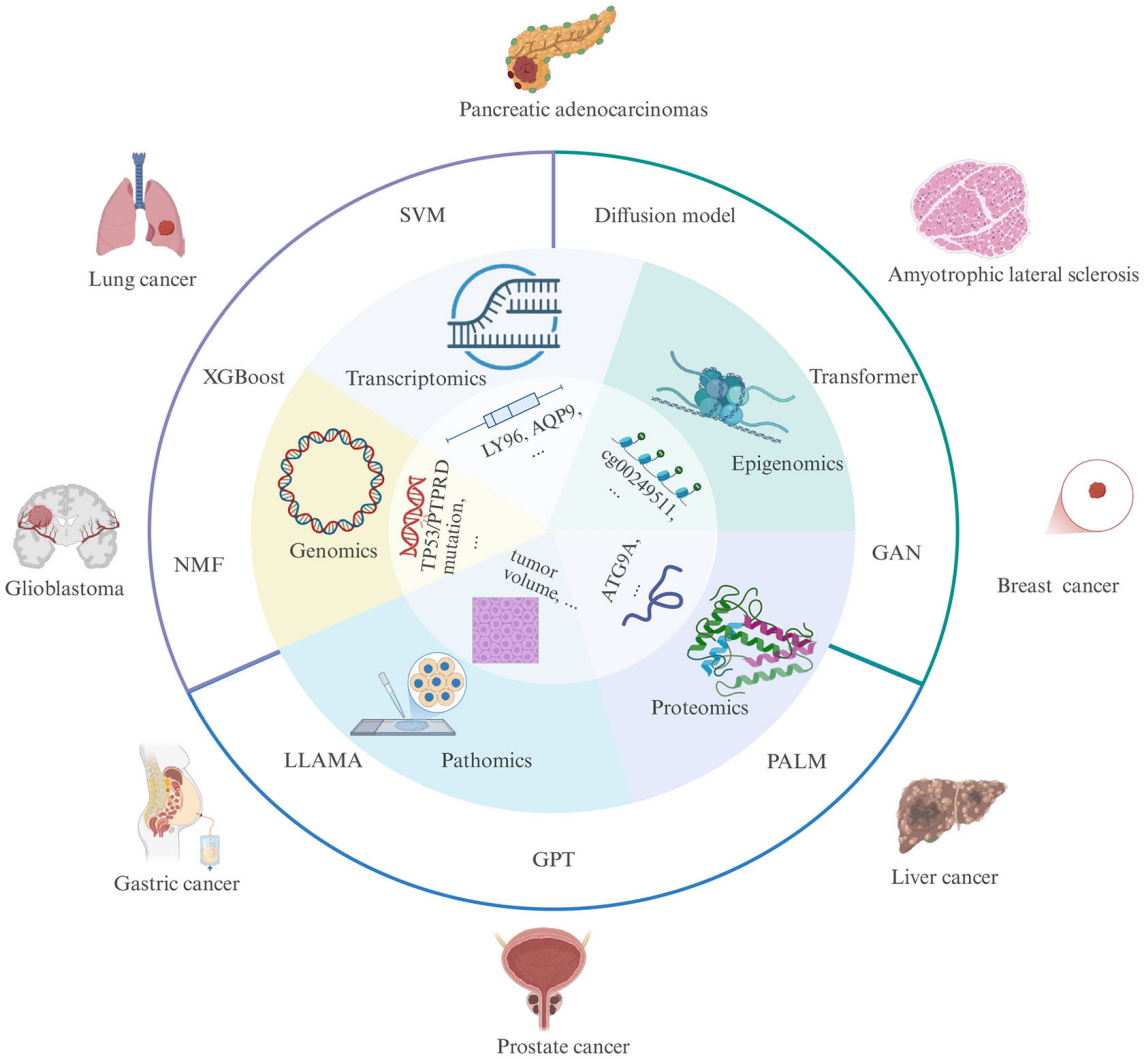
An overview for leveraging AI for biomarker identification in cancer immunotherapy. The outer sphere summarizes representative cancer types in which AI/ML approaches have been applied to identify diagnostic biomarkers or molecular signatures. The first layer groups diverse AI/ML models into three main categories: traditional machine learning models (e.g., SVM, XGBoost, VAE), deep learning models (e.g., transformer, diffusion model, GAN), and large language models (e.g., LLaMA, GPT, PaLM). The second layer depicts the major omics domains explored in AI/ML‐based biomarker discovery, encompassing genomics, transcriptomics, epigenomics, proteomics, and pathomics. The core of the sphere details a representative biomarker identified within each omics category. This figure is created with BioRender.com.

Recent advancements in AI offer novel and promising strategies to overcome these obstacles in cancer immunotherapy biomarker discovery (Figure 3). Specifically, heterogeneous omics data, including genomics, transcriptomics, epigenomics, proteomics, and EHR, can be leveraged to discover biomarkers that generalize across cancer types such as lung cancer, gastric cancer, and breast cancer. Three major model families dominate this landscape. Traditional ML models, including SVM, XGBoost, and NMF, remain widely used for interpretable, feature‐driven biomarker selection across structured multi‐omics datasets. Deep learning architectures, including transformer, diffusion model, and GAN, enable automatic feature extraction from high‐dimensional data such as histopathology images, RNA‐seq profiles, and proteomic spectra, revealing nonlinear molecular and spatial patterns associated with immune activity. LLMs, including LLaMA, GPT, and PaLM, extend this paradigm by integrating heterogeneous biomedical and clinical information, facilitating knowledge extraction and multimodal biomarker reasoning. Together, these models are applied to uncover reproducible diagnostic and prognostic signatures that support patient stratification and precision immunotherapy.

### 3.1 Transcriptomics‐Based Biomarker Identification

Transcriptomics focuses on profiling all RNA transcripts within cells to understand how gene activity changes under various biological or pathological conditions. It reveals dynamic molecular responses to disease progression, environmental stress, or therapeutic intervention. By characterizing these expression dynamics, transcriptomics provides immune response biomarkers and stratifies patients for immunotherapy treatment. To handle the high dimensionality and nonlinear structure of gene expression data, ML techniques have been widely applied in transcriptomics‐based biomarker identification for cancer immunotherapy. In transcriptomics, matrix factorization is widely used. NMF is a representative method that decomposes high‐dimensional profiles into a small set of additives, interpretable latent programs. For an omics data matrix $X\;\in \;R_{\geq 0}^{n\times p}$, where rows denote patients and columns denote molecular features, NMF seeks two non‐negative matrices $W\;\in \;R_{\geq 0}^{n\times k}$, and $H\in R_{\geq 0}^{k\times p}$ such that **X** ≈ **WH** by solving $\min_{W\geq 0,H\geq 0}{∥X-\mathrm{WH}∥}_{F}^{2}+\lambda_{W}\Omega \left(W\right)+\lambda_{H}\Omega \left(H\right)$. Here, rows of **H** define *k* “parts‐based” molecular programs (gene/protein signatures), and rows of **W** give patient‐specific program loadings, enabling subtype discovery by clustering patients in the *k*‐dimensional loading space. The non‐negativity constraint encourages additive mixtures that are often biologically plausible for processes such as immune infiltration or cell‐state composition, making NMF especially useful when interpretability is a primary goal. Key limitations include that NMF is sensitive to noise and batch effects, and it may fail when subtype differences depend on nonlinear interactions or negative correlations. For example, Luca et al. [99] developed EcoTyper to infer transcriptional cell states and higher‐order carcinoma ecotypes from bulk expression across 16 carcinomas. After estimating cell‐type‐resolved expression from bulk profiles, cell‐type‐specific NMF was applied to discover states. To construct ecotypes, EcoTyper aggregated across inferred states by quantifying state co‐occurrence across samples and clustering the resulting state–state network into recurrent multicellular communities. The learned state/ecotype representations can then be applied to independent cohorts by projecting new samples onto the learned basis to obtain state abundances. Using this workflow, 69 cell states and 10 carcinoma ecotypes were identified, and reported that the proinflammatory ecotype CE9 outperformed a panel of 112 biomarkers, including TMB, for predicting immunotherapy response. In addition, Zhu et al. [100] combined CoxBoost and Supervised Principal Components (SuperPC) to construct an epithelial cell marker gene prognostic signature (ECMGPS) for prostate cancer. Starting from 543 epithelial marker genes, CoxBoost selected 21 prognostic features that SuperPC condensed into a risk score. When benchmarking against standard‐of‐care clinical features, including PSA level, TNM staging, and Gleason score, ECMGPS demonstrated superior prognostic performance, achieving a higher concordance index and remaining an independent predictor in multivariate analyses. SuperPC, a supervised dimension‐reduction algorithm that first filtered genes by their correlation with outcome variables and then extracted principal components most predictive of survival, leading to more effective classification and regression. However, SuperPC used the outcome variable to guide feature selection and component derivation, which might lead to overfitting to random noise or dataset‐specific patterns in the training data. In another study, Ding et al. [101] proposed a hybrid pipeline integrating LASSO with survival‐SVM to derive a 17‐gene cell‐death signature (CDS) for LUAD. LASSO performed embedded feature selection by shrinking non‐informative coefficients to zero, and the retained signatures were modeled with survival‐SVM, which optimized pairwise risk ranking for censored data to capture non‐linear relations between expression and survival. Across multiple independent cohorts, CDS consistently showed a higher C‐index than routine clinical variables, including age, gender, and clinical stage, and remained prognostically significant after adjustment for these factors. However, this design combined LASSO with survival‐SVM might lead to the rapid burgeoning of its computational cost if the training size increases, limiting its scalability to very large datasets. Similarly, Zhang et al. [102] leveraged AI to elucidate the role of dendritic cells (DCs) in LUAD and their association with immunotherapy response. Univariate Cox regression identified 33 survival‐associated genes, which were further modeled using an integrated CoxBoost and elastic net framework to derive a seven‐gene prognostic signature defining DCIRS, a dendritic‐cell‐implicated risk model connecting DC‐related transcriptional activity to patient outcomes and immunotherapy response. DCIRS achieved time‐dependent AUCs of approximately 0.71 for 1‐year overall survival in the TCGA‐LUAD cohort. Across multiple independent datasets, DCIRS outperformed routine clinical variables such as age, gender, and TNM stage. The elastic net effectively handled multicollinearity by grouping correlated features and selecting the most representative ones. However, its performance might decline when the number of features is much smaller than the number of observations.

### 3.2 Epigenomics‐Based Biomarker Identification

Epigenomics is the comprehensive study of genome‐wide epigenetic modifications, which are reversible chemical change that regulates gene activity without altering the underlying DNA sequence. Epigenetic alterations play an important role in cancer and have emerged as promising therapeutic targets, including immunotherapy approaches that harness the immune system to eliminate tumor cells. These features make epigenomic markers highly sensitive indicators of cell state, disease progression, and therapeutic response. For epigenomic modeling, RF remains a practical choice for heterogeneous biomedical features. Conceptually, RF learns *B* decision trees ${\left\{T_{b}\right\}}_{b=1}^{B}$ using bootstrap‐resampled training sets and random feature subsets, then aggregates their predictions to reduce variance and improve generalization. For a sample *x*, the forest prediction can be written as an ensemble average. In classification, this is typically majority voting, $\hat{y}\;\left(x\right)=mode{\left\{T_{b}\left(x\right)\right\}}_{b=1}^{B}$, and in regression, it is the mean prediction, $\hat{f}\;\left(x\right)=\frac{1}{B}\;\sum_{b=1}^{B}T_{b}\left(x\right).$ Leveraging these properties, Pan et al. [103] defined LUAD immune “Hot” vs “Cold” immunophenotypes by computing ssGSEA‐based immune enrichment scores from five immune expression signatures and applying consensus clustering to derive immune subtypes, which were then consolidated into the two immunophenotypes. They next built an RF‐centered feature selection combined validation pipeline to derive the immunophenotype‐related methylation signature *(iPMS)* from immunophenotype‐specific differentially methylated CpG sites. Concretely, they repeatedly ran 10‐fold cross‐validation and, within each training fold, used mRMR to rank CpGs by mutual information, kept the top 10% as candidates, and trained an RF classifier; Performance was quantified by the balanced error rate (BER) on the held‐out fold and the entire CV procedure was repeated 1000 times with a randomized negative‐control to guard against chance learning. The signature size was fixed at five CpGs when BER no longer improved, and a representative 5‐CpG set was selected by minimizing its average distance to other candidate signatures. After deriving the 5‐CpG iPMS, they converted it into a continuous score by constructing an iPMS reference matrix and estimating sample‐level Hot/Cold mixture weights via deconvolution (RPC). This yielded 81.2% accuracy for immunophenotype assignment in the TCGA test split and showed consistent immune‐infiltration correlations across five external LUAD cohorts. Finally, in anti–PD‐1/PD‐L1–treated patients, their derived risk score achieved AUC = 0.752 for distinguishing durable from nondurable clinical benefit, outperforming established immunotherapy biomarkers such as TMB (AUC = 0.651) and aneuploidy score (AUC = 0.573).

### 3.3 Proteomics‐Based Biomarker Identification

Proteomics is the study of the entire set of proteins expressed by a cell, tissue, or organism at a specific time, aiming to characterize the composition, structure, abundance, post‐translational modifications, and interactions of proteins. Given that tumor antigens and immune checkpoint molecules are themselves peptides and proteins, it is unsurprising that proteomics has become a valuable tool for investigating tumor‐immune interactions and informing cancer immunotherapy research. A typical approach for proteomics is classical statistical feature selection, with forward stepwise variable selection offering a simple way to build an interpretable regression model using a small set of predictors. The method constructs a nested sequence of models by starting from an intercept‐only model and adding one variable at a time. At step *t*, given the currently selected set *S_t_*, it chooses the next variable by the greedy rule $j^{*}=\arg\max_{j\notin S_{t}}\in \Delta \left(S_{t},j\right)$, where Δ(*S_t_*,*j*) denotes the improvement in model fit obtained by adding variable *j* to the current model, such as the decrease in residual error. The approach is most useful when the sample size is limited and computational simplicity is important. However, because the procedure is greedy, it can miss the globally optimal feature set. For example, Mondelo‐Macía et al. [104] investigated whether pretreatment plasma proteomics can stratify NSCLC patients receiving first‐line pembrolizumab. Using SWATH‐MS in a discovery cohort, they first performed quality control and normalization, then identified differentially expressed proteins between responders (R) and non‐responders (NR). From the tighter differentially expressed proteins set (p < 0.01), they constructed a minimal predictive signature via stepwise forward variable selection with Akaike Information Criterion (AIC): starting from a null model, they iteratively added the single protein that produced the largest AIC improvement, yielding a nested sequence of models and stopping when further additions no longer improved AIC. This procedure selected a 7‐protein panel (ATG9A, DCDC2, HPS5, FIL1L, LZTL1, PGTA, SPTN2) for R/NR discrimination. The resulting predictive model demonstrated strong discriminative power, achieving an AUC of 1.0 in both discovery and validation cohorts, significantly outperforming PD‐L1 expression as a biomarker.

### 3.4 Pathomics‐Based Biomarker Identification

Pathomics is the large‐scale, systematic extraction of quantitative morphological features from histopathology images, typically derived from semantic segmentation of tissue and cellular structures, to create high‐dimensional datasets that enable computational pathology analysis and integration with other omics data. With the integration of AI, pathomics enables high‐throughput, automated feature extraction from WSI, enabling quantitative evaluation of the TME from histology slides. Pathomics is driven by CNN‐based models. For an input image *X*, a CNN layer computes a set of feature maps by convolution followed by a nonlinearity, *H* ^(*l*)^ =  σ (*W* ^(*l*)^*H ^(*l*1)^ + *b* ^(*l*)^), where *W* ^(*l*)^ and *b* ^(*l*)^ denote the learnable kernels and biases, respectively, *is the convolution operator, *H* ^(*l*)^ denotes the layer‐*l* feature map, and σ(·)is an activation function. Stacking layers yields progressively more abstract representations, and a task‐specific head produces a prediction $\hat{y}=g\left(H^{\left(L\right)}\right)$ that is trained by minimizing a supervised loss $\min_{\theta }\sum_{i}\ell \left({\hat{y}}_{i},y_{i}\right)$. CNNs are most beneficial when imaging data are readily available at scale and when slide‐ or patient‐level labels can be obtained, enabling models to exploit large cohorts for robust feature learning. However, performance can be sensitive to domain transformations such as staining protocols. Hu et al. [105] developed a two‐stage, supervised CNN workflow on H&E‐stained whole‐slide images from HR+/HER2− breast cancer patients to predict clinicopathological variables, multi‐omics events, immune‐related markers, and prognosis. WSIs were scanned and tessellated into 256×256 tiles after ROI selection and background filtering. Tiles were then passed through (i) a tile‐level tissue‐type classifier (ResNet‐18) to segment major tissue compartments and (ii) a second ResNet‐18 model trained on tissue‐relevant tiles for each prediction target. Patient labels were propagated to tiles during training, and inference used patient‐level score aggregation by averaging tile scores. For immunotherapy‐relevant readouts, the CNN predicted iTILs, sTILs, CD8A, and PDCD1 with test‐set AUCs in the 0.71–0.78 range. For prognosis, they compared a clinic‐only baseline against an integrated model that concatenated clinical covariates with deep tile features and trained a DeepSurv‐style survival network, reporting modest but consistent gains in C‐index for OS/RFS. To improve interpretability, the authors inspected high‐scoring tiles and used class activation maps to localize discriminative regions. A key limitation was that the pipeline was trained and tested within a single center without external validation, potentially reducing robustness under site/stain shift. Similarly, Dercle et al. [106] developed an RF survival model based on 25 imaging features derived from tumor segmentations in CT scans, aiming to identify a feature combination that could effectively predict overall survival (OS) in melanoma patients receiving pembrolizumab. The final model incorporated five variables: four imaging‐based and one clinical (treatment type). The imaging components included: (1) the change in total tumor volume over time, (2) baseline tumor volume, (3) changes in a feature (component 4) representing tumor spatial heterogeneity, and (4) changes in another feature (component 2) capturing the morphological characteristics of the tumor edges. When tested in an independent validation cohort of 287 patients, the model achieved an AUC of 0.92 (95% CI: 0.89–0.95) for OS prediction. When directly benchmarking against the current standard clinical decision rule, RECIST 1.1, the radiomics signature achieved substantially higher discriminative performance (AUC 0.92 vs. 0.80), highlighting that combining pathomics analysis with ML can enhance prognostic assessments using standard CT scans in the context of immunotherapy for melanoma. The RF approach offered strong performance on tabular pathomic data, but it may overfit with limited feature tuning.

### 3.5 Genomics‐Based Biomarker Identification

Genomics is the comprehensive study of the entire genome, aiming to understand the structure, function, and interactions of genes, as well as how genetic variations influence biological processes, traits, and diseases. Genomics guides immunotherapy by revealing tumor antigens, immune‐evasion mechanisms, and biomarkers that predict treatment response. The integration of ML with genomic profiling now enables the extraction of predictive mutation patterns directly from large‐scale sequencing datasets. Typical approaches for genomics are sparse linear modeling and margin‐based classifiers, with LASSO and SVM being two widely used methods for high‐dimensional omics data. For regression or risk modeling, LASSO estimates coefficients by minimizing a loss with an $\ell_{1}$ sparsity penalty, $\hat{\beta }=\;\arg\min_{\beta }\;\frac{1}{n}\sum_{i=1}^{n}\ell \;\left(y_{i},x_{i}^{⊤}\beta \right)+\lambda ∥\beta {∥}_{1}$, where *x_i_* denotes the omics feature for patient *i*, *y_i_* is the corresponding outcome, ***β*** is the coefficient vector, $\hat{\beta }$ is the fitted coefficients, and λ > 0 controls sparsity by driving many β_*j*_ to zero to yield a compact biomarker panel. For classification, SVM learns a decision function, *f*(*x*)  =  sign(*w* ^⊤^ *x* + *b*) by solving $\min_{w,b}\;\frac{1}{2}∥w{∥}^{2}+C\sum_{i=1}^{n}\xi_{i}$ subject to margin constraints, where ***w*** is the separating hyperplane normal vector, ***b*** is the intercept, ξ_*i*_ ≥ 0 slack variables that allow misclassification, and *C* > 0 trades off margin size against training errors. In practice, LASSO and SVM often outperform more flexible deep models when the sample size is limited and when the signal is approximately linear. However, LASSO can be unstable under strong multicollinearity, while SVM performance is sensitive to kernel choice and hyperparameter tuning. For a detailed example of combining LASSO and SVM in practice, Peng et al. [107] analyzed somatic mutation profiles from EGFR/ALK‐negative non‐squamous NSCLC patients treated with ICIs, using 83 patients (CheckMate‐012 + DFCI) as a training cohort and 165 patients (MSKCC) as an independent validation cohort. They encoded each patient's tumor as a high‐dimensional binary mutation vector derived from WES (training) or targeted NGS (validation), and then implemented a two‐step modeling pipeline: (i) 5‐fold cross‐validated LASSO to select a compact gene panel by shrinking non‐informative coefficients to zero, yielding a 15‐gene SMS; (ii) an SVM classifier trained on this reduced feature set to predict best overall response (BOR) defined by RECIST 1.1, with the classification threshold chosen using the maximum Youden index on the ROC curve. In both training and validation sets, this SMS model demonstrated strong predictive accuracy (AUC = 0.859), outperforming commonly used biomarkers such as TP53 mutations, TMB, and PD‐L1 expression. Beyond response prediction, they also stratified patients into SMS‐high vs SMS‐low and showed significantly worse PFS/OS in SMS‐high, while multivariate Cox models supported SMS as an independent prognostic factor. Methodologically, this was a clear, actionable template for small‐to‐moderate genomic cohorts: LASSO enforced sparsity for a deployable gene panel, while SVM provided a margin‐based decision rule that can generalize under high dimensionality. However, the framework remained limited in capturing higher‐order epistatic interactions unless extended with interaction features, nonlinear kernels, or ensemble/DL alternatives, and it relied on retrospective cohorts with cross‐platform sequencing differences that warranted prospective, multi‐center validation.

### 3.6 EHR‐Based Biomarker Identification

EHRs are digital, longitudinal repositories of clinical data that capture the full scope of patients’ medical history, including structured information such as demographics, laboratory results, and medications, as well as unstructured information such as clinical notes, imaging reports, and pathology narratives. By leveraging diverse clinical information, EHR‐based analyses can reveal associations between patient characteristics, disease trajectories, and therapeutic outcomes, thereby supporting precision medicine. In EHR‐based biomarker identification, the central methodological challenge lies in extracting clinically meaningful patterns from heterogeneous, high‐dimensional data that encompass both structured variables and unstructured clinical narratives. To address this limitation, recent ML framework has been applied to enable automated phenotype discovery that aligns with biological or clinical relevance. For EHR data, latent‐variable generative modelings are commonly used, with the VAE, providing a principled way to learn low‐dimensional, denoised representations from high‐dimensional omics profiles. Given an input sample ***x*** such as a gene‐expression vector, a VAE assumes a generative process in which a latent variable ***z*** is first drawn from a simple prior *p*(*z*), and the observed data are generated from a decoder distribution *p*_θ_(*x*∣*z*). Because the true posterior *p*(*z*∣*x*)is intractable in neural decoders, the VAE introduces an encoder *q*_ϕ_(*z*∣*x*) EHRto approximate it and optimize the evidence lower bound, $\log p_{\theta }\left(x\right)\;\geq \;E_{q_{\phi }\left(z|x\right)}\left[\log p_{\theta }\left(x|z\right)\right]-\mathrm{KL}\;\left(q_{\phi }\left(z|x\right)\;∥\;p\left(z\right)\right)$. This objective makes explicit the two forces that shape the learned embedding: the reconstruction term encourages *z* to preserve information needed to regenerate the input, while the KL term regularizes *q*_ϕ_(*z*∣*x*) toward the prior, producing a structured latent space that supports interpolation and robustness to noise. VAEs are most beneficial in settings with noisy or partially missing omics measurements. However, performance depends on the assumed likelihood model and the strength of KL regularization, and overly strong regularization can yield posterior collapse and under‐informative embeddings. For instance, Wang et al. [108] proposed GETM (graph‐embedded topic model) to learn interpretable patient phenotypes from sparse, multimodal EHR‐style records by injecting biomedical taxonomy structure into an ETM‐style VAE topic model. Concretely, they first constructed two graphs that encoded condition taxonomy and medication taxonomy (e.g., tree‐structured hierarchies) and ran node2vec to obtain fixed embeddings for each condition and medication node. These embeddings defined a structured feature space in which related codes were already close. GETM then trains an ETM‐like generative model over two bag‐of‐words matrices: a VAE encoder mapped each patient's binary/count feature vector to a logistic‐normal topic mixture (patient proportions over K latent phenotypes), while two linear decoders reconstructed condition and medication distributions from the shared topic mixture using modality‐specific topic embeddings. This design made topic semantics stable and clinically coherent because topic‐feature associations were anchored by taxonomy‐informed node embeddings, improving both imputation and phenotype interpretability relative to ETM without graph information.

### 3.7 Multi‐omics Based Biomarker Identification

In clinical practice, panels of molecular biomarkers offer an efficient and cost‐effective means of predicting patient prognosis [109]. Yet single‐omics approaches seldom capture the full complexity of the TME. Signals detected in one layer, for instance, gene expression changes can be dampened or magnified by another promoter methylation. As a result, single‐omics snapshots may overlook critical causal links and yield brittle predictors. By contrast, AI‐driven integration of multi‐omics profiles synthesizes genomics, epigenomics, transcriptomics, proteomics, and pathomics data, providing a deeper, more robust understanding of tumor‐immune dynamics and enabling the discovery of clinically actionable biomarkers [110]. Multi‐omics AI frameworks for biomarker discovery can be categorized into two architectural paradigms: (1) *feature‐integrated ML*, (2) *modality‐specific DL*.

*Feature‐integrated ML* explicates feature selection and dimensionality reduction techniques followed by traditional ML classifiers. Han et al. [111] developed an integrative LUAD framework combining iClusterBayes for multi‐omics fusion with RSF and survival‐SVM for prognostic modeling. Their consensus ML‐derived risk signature (CMRS), composed of 13 genes, successfully delineated two molecular LUAD subtypes with distinct immune and clinical features. Similarly, Wang et al. [112] constructed a TCGA LUAD multi‐omics predictor of TMBß by selecting seven genes, seven miRNAs, and six CpG sites from differentially expressed features, yielding AUCs of 0.911 and 0.859 in training and validation cohorts, respectively. The model's strong correlation with ground truth TMB suggested its utility as a non‐invasive surrogate, potentially streamlining patient stratification for immunotherapy. More recent studies have extended classical ML feature engineering to integrative and spatial frameworks. Mao et al. [113] derived a mitotic network activity index (MNAI) based on 54 mitotic apparatus genes, showing that high MNAI predicted benefit from anti‐PD‐1/CTLA‐4 therapies. When combined with AI‐derived cellular morphometric subtypes from histological imaging, predictive performance further improved, surpassing either biomarker alone. Likewise, Che et al. [114] demonstrated the power of spatial multi‐omics integration in gastric cancer by combining single‐cell RNA sequencing, multiplex immunohistochemistry, and spatial transcriptomics. They trained an SVM using spatial and molecular metrics, achieving an AUC of 0.84 for predicting combined fluorouracil/oxaliplatin and anti‐PD‐1 therapy response.

*Modality‐specific DL* leverages DNN to extract rich representations from individual data modalities, such as autoencoders for molecular profiles, followed by integration strategies that combine these learned features for biomarker prediction. At the molecular scale, Wang et al. [115] proposed *FactVAE*, a factorized VAE framework for paired scRNA‐scATAC integration that targeted two practical outputs at once: (i) high‐quality cell embeddings for clustering/visualization and (ii) feature embeddings that support regulatory (gene–peak) inference. FactVAE trained two modality‐specific VAEs (RNA‐VAE and ATAC‐VAE). The VAE was applied in the usual “encode, sample, decode” loop, but with a crucial twist in decoding: instead of a generic neural decoder, FactVAE used a factorized decoder where cell embeddings ***z*** were multiplied by trainable feature‐embedding matrices (genes/peaks) to parameterize a ZINB likelihood for reconstruction. For ATAC, the decoder produced ZINB parameters through matrix products, alongside dispersion and zero‐inflation terms from separate factor matrices, which made the learned peak embeddings explicitly interpretable and reusable. Training was organized as a two‐stage procedure: (1) a joint phase that co‐optimized RNA‐VAE and ATAC‐VAE while adding a regulatory constraint that aligned gene and peak embeddings to known gene–peak, and (2) an ATAC‐focused phase that applied similarity structure alignment (SSA) to transfer cell‐geometry knowledge from RNA to ATAC, improving ATAC representations and enabling ATAC data augmentation. Empirically, the authors reported stronger multi‐omics clustering performance than multiple baselines across diverse protocols, clearer cell‐type‐specific motif signals after augmentation, and improved gene–peak regulatory inference when benchmarked against external resources such as eQTL and pcHi‐C. Moving toward multimodal fusion, Qie et al. [116] developed a DL framework that integrated H&E WSI with mRNA, lncRNA, DNA methylation, and copy‐number variation from TCGA colorectal cancer samples. A CNN trained on histology tiles achieved an AUC of 0.809, which increased to 0.952 after compacting bilinear pooling of image and molecular features, underscoring the power of multimodal integration for MSI prediction. The study also cautioned that unrefined fusion may degrade performance, highlighting the importance of modality‐specific preprocessing and feature selection pipelines. Expanding the modality repertoire, Tong et al. [117] integrated tissue microbiome profiles, bulk RNA‐seq immune deconvolution scores, and histopathology images from 1,324 NSCLC cases. Their DL classifier, which appended latent microbiome and transcriptome embeddings to histology features, predicted TP53 mutation status with an AUC of 0.84, revealing a microbiome‐immune‐histology continuum that could inform microbial biomarker discovery. Collectively, these studies demonstrated that modality‐specific DL architectures enabled the automatic discovery of complex, high‐dimensional patterns within each data type, substantially reducing reliance on manual feature engineering. However, the separate processing of each modality followed by fusion may fail to capture synergistic interactions that emerged only when data types were jointly modeled.

The reviewed studies demonstrate that AI has significant utility in biomarker discovery for cancer immunotherapy. Across omics‐driven immunotherapy studies, the models largely fall into four complementary paradigms with distinct trade‐offs. *Conventional statistical and ML methods* (e.g., CoxPH/CoxBoost, LASSO/elastic net, SVM, RF/RSF, and stepwise selection) remain strong baselines because they are relatively robust, computationally efficient, and often easier to interpret, making them well‐suited for small‐to‐moderate cohorts where clinical transparency and deployable biomarker panels are priorities. However, these approaches may struggle to capture complex nonlinear effects, higher‐order feature interactions, and cross‐modality dependencies. *Unsupervised representation methods* such as NMF provide biologically intuitive, parts‐based decompositions that are valuable for tumor microenvironment state discovery and subtype/ecotype characterization, but they can be sensitive to rank choice, initialization, and batch effects. *Deep learning models* (e.g., CNN, DeepSurv, neural networks) offer strong capacity to learn hierarchical features and nonlinear risk functions from images or high‐dimensional profiles, which can improve stratification when data scale is sufficient, yet they face practical barriers including domain shift across centers, limited interpretability, and increased overfitting risk in small or imbalanced datasets. Finally, *VAE‐based generative models* (e.g., VAE, ETM‐style models) provide a principled way to learn denoised low‐dimensional patient phenotypes, handle sparsity or missingness, and inject structured knowledge from biomedical ontologies, which is particularly useful for EHR phenotyping and multi‐omics alignment, but performance depends on embedding quality, modeling assumptions, and careful external validation. In practice, ML/DL models can process vast and complex datasets, such as EHR and multi‐omics profiles, that often remain underutilized by conventional analytic methods. By leveraging the high dimensionality and heterogeneity of these data, AI enables the identification of clinically relevant molecular features linked to diagnosis, prognosis, treatment response, and immune phenotypes. This capacity allows AI not only to improve predictive accuracy but also to reveal novel biomarker signatures that may be overlooked by traditional statistical approaches. These advances strengthen the transformative role of AI in precision oncology and its promising potential in guiding immunotherapy decisions through robust biomarker selection.

Detailed comparisons of different types of approaches for biomarker identification in cancer immunotherapy are summarized in Table 4. Across these studies, risk‐score and sparse feature–selection pipelines (e.g., Cox‐based models, LASSO/elastic net, and stepwise selection) are valued for their clinical interpretability and simple deployment, but they can be unstable under noise and correlated features and often miss complex nonlinear effects. Kernel and ensemble methods (e.g., SVM, random forest/survival forest) typically offer stronger flexibility for high‐dimensional tabular data and can capture some nonlinear patterns, yet they may incur higher computational cost and still overfit without careful tuning, with interpretability remaining limited. Deep learning approaches (e.g., CNN/DeepSurv and other neural networks) reduce reliance on manual feature engineering and enable cross‐modal representation learning, but they often face generalizability challenges across cohorts/sites. Different omics (including transcriptomics, epigenomics, proteomics, pathomics, genomics, EHR, and multi‐omics), key ideas, key features, years, and references are elaborated.

**TABLE 4:** A summary of AI models developed for biomarker identification in cancer immunotherapy. Studies use AI/ML models to identify biomarkers for immunotherapy response prediction in cancers. The table includes omics types, algorithms used in the references, key features, and reference sources. Algorithms are listed according to the omics type.

Omics	Method	Key ideas	Year	References	Key features and limitations
Transcriptomics	ECMGPS	CoxBoost and SuperPC	2023	Zhu et al. [100]	Stable and robust; Overfitting to random noise
	CDS	Lasso and survival‐SVM	2023	Ding et al. [101]	Handles complex patterns; High computational cost
	DCIRS	CoxBoost and Enet	2023	Zhang et al. [102]	Interpretable risk score; Limited nonlinear interactions
	EcoTyper	NMF	2021	Luca et al. [99]	Multi‐cell‐type state discovery; Unstable in noisy data
Epigenomics	iPMS	RF, minimum redundancy feature selection	2022	Pan et al. [103]	Simple and transparent; Rely on preselected features
Proteomics	SFVS	Stepwise forward variable selection	2024	Mondelo‐Macía et al. [104]	Interpretable; Unstable and prone to overfitting
Pathomics	DeepSurv	CNN, DeepSurv	2023	Hu et al. [105]	No manual feature engineering; Limited generalizability
	RFSurv	RF	2022	Dercle et al. [106]	Robust to mixed features; Overfitting without tuning
Genomics	SMS	LASSO, SVM	2022	Peng et al. [107]	Good for high‐dimensional data; Limited nonlinear modeling
EHR	GETM	VAE and node2vec	2022	Wang et al. [108]	Better imputation; Rely on pretrained embeddings
Multi‐omics	CMRS	Random survival forest, survival‐SVM	2024	Han et al. [111]	Captures nonlinear survival patterns; Limited interpretability
	TPM	LASSO	2022	Wang et al. [112]	Sparse feature selection; Limited generalizability
	MNAI	CoxPH	2023	Mao et al. [113]	Interpretable, simple score; Lack of validation
	Spatial‐SVM	SVM	2024	Che et al. [114]	Suitable for high‐dimensional features; Limited nonlinear modeling
	FactVAE	VAE	2025	Wang et al. [115]	Handles ATAC sparsity; Struggling with unpaired multi‐omics
	MSI‐CNN	CNN	2022	Qie et al. [116]	Captures cross‐modal relations; Limited interpretability
	Tri‐Modal‐NN	Neural network	2024	Tong et al. [117]	Learns pathology features automatically; Limited generalizability

Abbreviations: NMF, non‐negative matrix factorization; VAE, variational autoencoder; LASSO, least absolute shrinkage and selection operator; RF, random forest; XGBoost, eXtreme gradient boosting; ETM, embedded topic model; SVM, support vector machine; Enet, elastic network; CNN, convolutional neural network; RSF, random survival forest; MLP, multilayer perceptron; SuperPC, super point cloud.; MNAI, mitotic network activity index. For ease of reference, we have coined the method names for those references that do not provide their method names.

## 4 AI for Treatment Strategy Optimization in Cancer Immunotherapy

**FIGURE 4:**
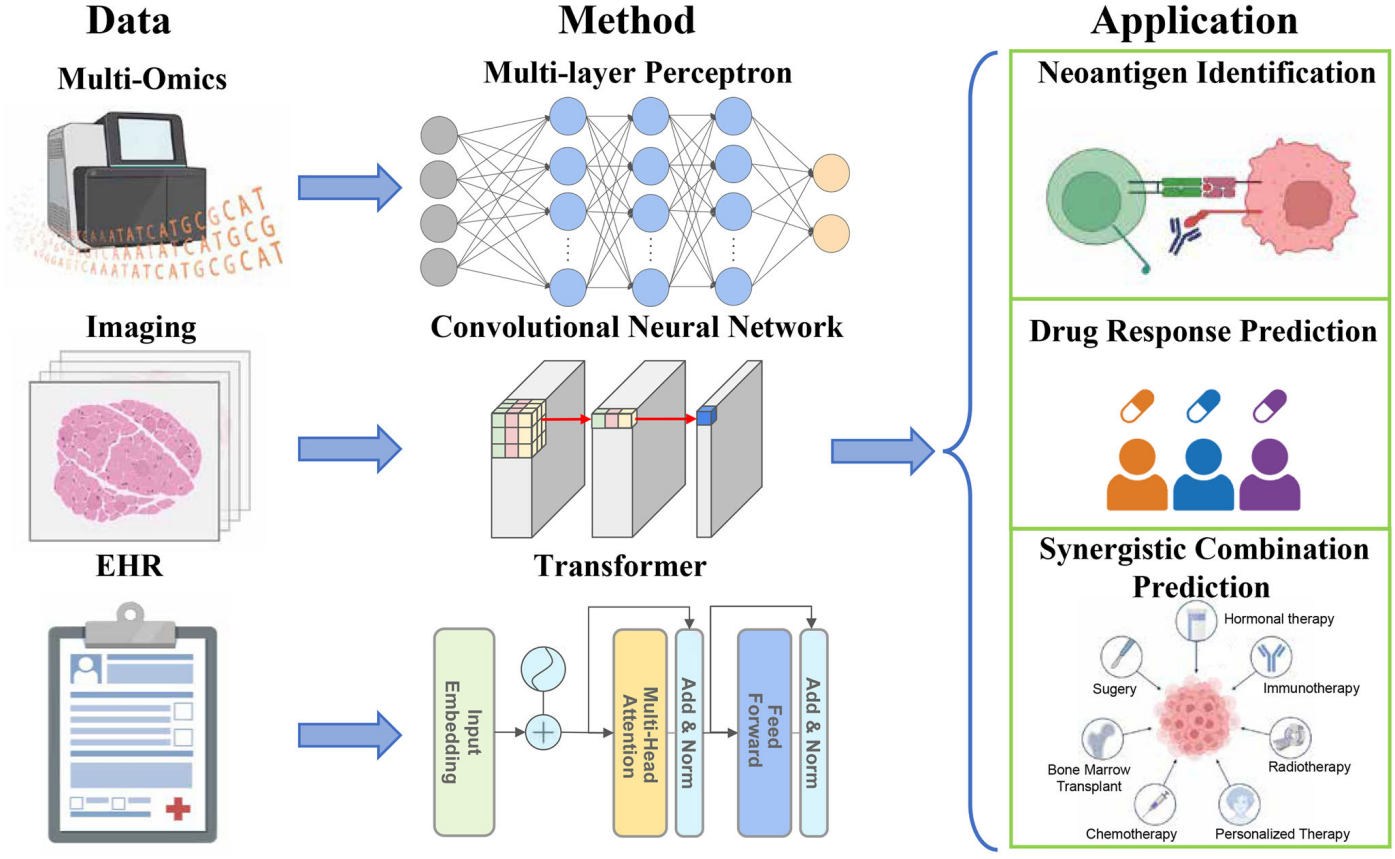
A typical workflow for leveraging AI for treatment strategy optimization in cancer immunotherapy. Multi‐omics, imaging, and electronic health record (EHR) data can be analyzed using AI models such as multilayer perceptron (MLP), convolutional neural networks (CNN), and transformers. These AI‐driven approaches facilitate critical applications, including neoantigen identification, prediction of patient‐specific drug response, and optimization of synergistic therapeutic combinations to improve personalized immunotherapy strategies.

Treatment strategy is the comprehensive plan for controlling a patient's disease through therapeutic interventions to achieve optimal clinical outcomes. It encompasses multiple clinical decisions, including drug selection, treatment scheduling, dosing regimen, and combination design [118]. Treatment strategy optimization is the systematic process of refining these interventions to enhance therapeutic efficacy and patient outcomes. The optimization of treatment strategies is at the core of cancer immunotherapy, as clinical outcomes depend on the coordination of therapeutic agents, dosing regimens, and treatment timing to elicit effective and durable antitumor immune responses. The complexity of cancer immunotherapy presents unprecedented challenges for treatment optimization, driven by intricate interactions among tumor biology, immune dynamics, patient heterogeneity, and therapeutic factors. Addressing these challenges requires accurate prediction of patient response, identification of synergistic drug combinations to overcome resistance, and personalization of treatment timing and dosing. Conventional treatment design has largely relied on empirical trial‐and‐error approaches, which are often limited in capturing the nonlinear and high‐dimensional nature of tumor‐immune interactions. By integrating diverse data modalities, including multi‐omics, imaging, and EHR, AI enables a comprehensive characterization of tumor‐immune interactions and therapeutic responses. This section details how AI is being applied across three key domains of treatment optimization: neoantigen identification, drug response prediction, and synergistic treatment prediction. A typical workflow (Figure 4) for treatment strategy optimization in cancer immunotherapy is detailed as follows. Specifically, multi‐omics profiles (e.g., genomic, transcriptomic, and proteomic data) are typically analyzed using architectures such as MLPs and GNNs to capture molecular interactions underlying therapeutic response. Imaging data, including histopathology or radiology, are most commonly processed by CNNs to extract spatial and morphological features associated with immune response and tumor heterogeneity. Meanwhile, EHRs are modeled using natural language processing (NLP) or transformer‐based architectures to learn temporal and contextual clinical patterns that influence patient outcomes.

### 4.1 Neoantigens Identification

Neoantigens are novel peptide antigens that arise from tumor‐specific alterations, including somatic mutations, aberrant RNA splicing, abnormal post‐translational modifications, or the expression of viral open reading frames integrated into the host genome [119]. Neoantigens are vital for triggering anti‐tumor immune responses and hold significant promise as targets for personalized cancer immunotherapy [119, 120]. The accurate identification of these neoantigens from a large pool of candidates is essential for developing effective treatments. Traditional approaches rely on genomic and transcriptomic sequencing combined with immunological assays, yet these methods face challenges in scalability, accuracy, and generalizability across diverse patient populations. More recently, computational methods, including *tree‐based ensemble methods*, *NLP‐based models*, and *Graph‐based models*, have been applied to identify and predict neoantigens.

*Tree‐based ensemble methods* combine multiple decision trees to improve predictive accuracy and robustness, commonly exemplified by models such as Random Forest, Gradient Boosting, and XGBoost. As a representative method, XGBoost is a scalable and regularized tree‐based ensemble learning method that has been widely adopted in biomedical prediction tasks. XGBoost builds an ensemble of decision trees, where each new tree is trained to correct the residual errors of the existing ensemble, enabling the model to capture complex nonlinear relationships and feature interactions while maintaining robustness to noise. XGBoost models the prediction for a sample *x_i_* as an additive combination of *K* regression trees: $\hat{y_{i}}=\sum_{k=1}^{K}f_{k}\left(x_{i}\right)\;,\;f_{k}\in F$. The F denotes the space of decision trees, and each tree *f_k_* maps an input sample to a leaf score. The *tree‐based ensemble methods* have significantly improved the prediction and prioritization of immunogenic neoantigens by integrating multi‐omics data, modeling peptide‐MHC binding interactions, and assessing T cell receptor recognition. A novel ranking algorithm [121] was introduced for class I candidate neoepitopes using next‐generation sequencing data and a dataset of 185 neoepitopes known to be recognized by HLA class I‐restricted TILs from metastatic cancer patients. Through a random forest model, researchers found that incorporating multiple factors influencing epitope presentation and recognition significantly improved the algorithm's sensitivity and specificity compared to relying solely on predicted HLA binding. Beyond commonly used features for neoantigen prioritization, Müller et al. [122] revealed that factors such as the location of neo‐peptides within protein HLA presentation hotspots, binding promiscuity, and the oncogenic role of the mutated gene were highly predictive for immunogenicity. The developed ML classifiers (logistic regression, SMV, XGBoost, CatBoost) accurately predicted neoantigen immunogenicity across datasets and improved ranking performance by up to 30%. In addition, ImmuneMirror [123], a balanced random forest machine learning‐based integrative pipeline and web server, was designed for predicting and prioritizing neoantigens. Furthermore, a 26‐gene XGBoost model [124] was developed to identify neoantigen‐reactive T cells directly from single‐cell transcriptomes of tumor‐infiltrating lymphocytes. Crucially, they found a significant positive correlation between the fraction of Neo T cells and clinical benefit and overall survival in patients undergoing neoadjuvant immunotherapy. To prioritize the neo‐peptides for immunotherapy, MaNeo [125], an immunopeptidomic‐guided machine learning‐based screening pipeline, was proposed. MaNeo proved highly accurate in identifying both shared and tumor‐specific canonical and noncanonical neo‐peptides. Furthermore, Vasudevan et al. [126] established a machine learning‐driven framework for identifying tumor antigens and immune targets, providing a strategy for colorectal cancer immunotherapy tailored to specific immune subtype responses. Machine learning models like LightGBM, XGBoost, and XGBRF were employed to predict optimal immune targets for vaccine design. These *tree‐based ensemble methods* provide interpretability through feature importance analyses, enabling researchers to uncover biologically meaningful predictors. However, *tree‐based ensemble methods* rely on manually engineered features derived from peptide sequences, expression profiles, or molecular descriptors. This dependence on handcrafted features restricts their ability to capture complex, nonlinear biological relationships inherent in tumor‐immune interactions.

The NLP focuses on the interaction between computers and human language, aiming to enable machines to read, understand, and derive meaningful insights from text or speech data. By inspirated from language modeling, the *NLP‐based models* have emerged as powerful tools for analyzing and interpreting biological sequences and biomedical text in cancer immunotherapy. These approaches treat amino acid or nucleotide sequences as “biological sentences”, enabling the extraction of contextual and semantic information embedded within peptide or protein structures. For example, DeepNeoAGNet [127], a novel sequence DL framework, was developed using bidirectional long‐short term memory (Bi‐LSTM) neural networks to extract both global and local bioengineering information from peptide sequences. Bi‐LSTM is an extension of the standard LSTM architecture designed to model contextual dependencies in sequential data by processing the sequence in both forward and backward directions. Bi‐LSTMs jointly model forward and backward sequence dependencies using gated memory mechanisms, enabling context‐aware representations that capture both upstream and downstream influences within biological sequences. DeepNeoAGNet incorporated comprehensive amino acid feature engineering, including global and local amino acid composition, dipeptide composition, and inter‐residue sequence relationship analysis to capture intricate dependencies within sequences. Most recently, Cai et al. [128] introduced CNNeo, a novel DL‐based neoantigen prediction model, and CNNeoPP, an integrated computational pipeline for neoantigen discovery. CNNeo leveraged NLP‐based sequence encoding and multi‐modal feature integration, achieving superior predictive performance over existing tools. CNNeoPP was thoroughly validated with independent datasets, including the TESLA dataset, and through experimental ELISpot T‐cell assays. Recognizing that optimal neoepitope selection for immune response elicitation is traditionally time‐consuming and costly, NEO [129] employed a sophisticated stacking ensemble method that integrates scores from state‐of‐the‐art models (MHCFlurry 1.6, NetMHCstabpan 1.0, and IEDB) using next‐generation sequencing data. The innovative architecture combined Feed Forward Neural Networks (FFNN) and RNN with LSTM layers, enabling comprehensive analysis of both sequential and non‐sequential data, with results from both models aggregated to produce highly accurate predictions. These *NLP‐based models* excel at learning context‐dependent relationships within peptide sequences, enabling the extraction of both global compositional patterns and local residue interactions. On the other hand, LSTMs, including bidirectional variants, struggle to capture long‐range dependencies and higher‐order structural relationships inherent in protein and peptide sequences. This limits their ability to fully represent spatial and conformational features for antigen presentation and immune recognition.

The *Graph‐based models* enable the capture of structural, spatial, and relational information critical for modeling molecular interactions. In *Graph‐based models*, biological entities such as proteins, peptides, or small molecules are represented as nodes, while their interactions, such as chemical bonds, binding affinities, or functional associations, form edges. For instance, GraphMHC [130], a novel model that leveraged GNN applied to molecular structures, was proposed to simulate the binding between MHC proteins and peptide sequences. The core of GNNs is message passing, in which each node iteratively updates its representation by aggregating information from its neighbors using normalized adjacency information: $H^{\left(l+1\right)}=\sigma \left({\tilde{D}}^{-\frac{1}{2}}\;\tilde{A}{\tilde{D}}^{-\frac{1}{2}}H^{\left(l\right)}W^{\left(l\right)}\right)$. The $\tilde{A}$ is the adjacency matrix with self‐loops added, $\tilde{D}$ is the corresponding degree matrix, **H** ^(*l*)^ is the matrix of node embeddings at layer *l*, **W** ^(*l*)^ is trainable weight matrix, and σ(⋅) denotes a nonlinear activation function. GraphMHC converted amino acid sequences from the Immune Epitope Database (IEDB) into molecular structures, extracting atomic intrinsic information and inter‐atomic connections to form a graph representation. The network, composed of stacked graph attention and convolution layers, achieved a high performance in predicting MHC‐peptide binding. GNN explicitly models the physical chemistry underlying binding interactions rather than relying solely on statistical sequence patterns and provides mechanistic insights by identifying which molecular substructures contribute most to binding affinity. However, generating accurate three‐dimensional molecular structures from sequences requires computational modeling, introducing additional uncertainty and computational cost.

Overall, these AI‐driven methods provide robust, scalable solutions for personalized cancer vaccine development, adoptive T‐cell therapy design, and pan‐cancer immunotherapy strategies, offering more accurate and clinically actionable neoantigen profiles than traditional approaches (Table 5). Detailed comparisons of different types of approaches for neoantigen identification in cancer immunotherapy are summarized in Table 5, categorized by model architecture into *Tree‐based ensemble methods*, *NLP‐based models*, and *Graph‐based models*. *Tree‐based ensemble methods* primarily rely on manually engineered features derived from peptide sequences, MHC binding affinity, and biochemical properties. Methods such as Neoepitope Ranking, ImmuneMirror, and MaNeo demonstrated strong interpretability and robust performance on structured tabular data. These models are particularly well‐suited for scenarios involving structured tabular data, limited sample sizes, and a strong need for interpretability. However, their reliance on predefined features limits their ability to capture higher‐order sequence dependencies and structural interactions. *NLP‐based models*, such as DeepNeoAGNet, CNNeoPP, and NEO, improve representation by modeling sequential dependencies and integrating multimodal features. However, these models often struggle with long‐range dependencies and lack explicit incorporation of three‐dimensional structural or physicochemical constraints. *Graph‐based models*, such as GraphMHC, encode molecular structures and physicochemical interactions using graph neural networks. By modeling residues and their interactions as graph entities, these methods provide a more mechanistic representation of peptide‐MHC binding. Despite improved biological fidelity, graph‐based approaches typically have higher computational costs and may introduce uncertainty due to limited structural data availability. In practice, the choice of model architecture should be guided by data availability, task scale, and interpretability requirements, with *tree‐based ensemble methods* favored for tasks requiring clinical transparency, *NLP‐based models* for those focusing on sequence‐driven scalability, and *Graph‐based models* for those emphasizing structure‐informed mechanistic insight.

**TABLE 5:** A summary of AI models developed for neoantigen identification. Models are listed chronologically within model categories. The Key Features and Limitations column presents the advantages and disadvantages of each approach category in “A; B” format, where A represents the advantages and B represents the disadvantages.

Model type	Method	Key ideas	Year	References	Key features and limitations
Tree‐based ensemble methods	Neoepitope Ranking	RF	2021	Gartner et al. [121]	Interpretability: Rely on manually engineered features
	Immunogenic Neoantigen Prediction	LR, SMV, XGBoost, CatBoost	2023	Müller et al. [122]	
	ImmuneMirror	Balanced RF	2024	Chuwdhury et al. [123]	
	Identification of neoantigen‐reactive CD8 + T cells	XGBoost	2024	Sun et al. [124]	
	MaNeo	RF and XGBoost	2025	Cai et al. [125]	
	AI‐driven Immune Subtyping	LightGBM, XGBoost, XGBRF	2025	Vasudevan et al. [126]	
NLP‐based models	DeepNeoAGNet	Bi‐LSTM	2024	Liu et al. [127]	Learning context‐dependent relationships; Struggle to capture long‐range dependencies and higher‐order structural relationships
	CNNeoPP	NLP‐based sequence encoding + multi‐modal feature integration	2025	Cai et al. [128]	
	NEO	FFNN + LSTM stacking	2024	Basava et al. [129]	
Graph‐based models	GraphMHC	Graph neural network	2024	Jeong et al. [130]	Models the physical chemistry underlying binding interactions; Uncertainty and computational cost

Abbreviations: RF, random forest; LR, logistic regression; DNN: Deep neural network; FFNN: Feed forward neural networks; RNN: Recurrent neural networks; LSTM: Long‐short term memory; NLP: Natural language processing.

### 4.2 Drug Response Prediction

Drug response prediction is to estimate how a specific drug will affect a particular biological system. In the context of cancer immunotherapy, it focuses on forecasting how a patient's tumor and immune system will respond to immunotherapies. Predicting how individual patients or cancer cell lines will respond to specific therapeutic agents remains one of the most challenging and clinically important problems in precision oncology [131]. Recent advancements in AI have significantly transformed the field of drug response prediction by enabling more precise and personalized therapeutic strategies in cancer treatment. In particular, DL models that incorporate multi‐omics data, chemical structures, and biological networks are being actively developed to improve the accuracy and interpretability of response predictions. These DL models can be broadly classified into *Graph‐based models*, *Hybrid Graph models*, and *Knowledge‐Guided DL*.

*Graph‐based models* have emerged as powerful tools for learning from structured biological data, such as molecular graphs and gene networks for drug response prediction. For example, Nguyen et al. [132] introduced GraphDRP, a novel method utilizing GCN for drug response prediction. In GraphDRP, drugs were directly represented as molecular graphs that capture atomic bonds, while cell lines were depicted as binary vectors of genomic aberrations. GCN layers learned representative features for both drugs and cell lines, which are then combined to characterize each drug‐cell line pair. A fully connected neural network then predicted the drug response value. Similarly, GraphCDR [133], a novel GNN method, was introduced to predict how cancer cell lines respond to therapeutic drugs. GraphCDR constructed a GNN by incorporating multi‐omics data from cancer cell lines and the chemical structures of drugs, along with known cancer drug responses. It also employed a contrastive learning task as a regularizer within a multi‐task learning paradigm to significantly enhance its generalization ability. By representing drugs as molecular graphs, GNNs can explicitly capture atomic connectivity, bond interactions, and higher‐order chemical features that are often lost in traditional vectorized representations. This structural awareness allows models to learn more biologically meaningful drug representations and improve generalization across unseen compounds. However, their performance is highly dependent on graph construction quality, where missing or noisy molecular or omics relationships can propagate errors through the network. Additionally, GNNs often struggle with scalability and computational efficiency when applied to large molecular graphs or high‐dimensional multi‐omics data.

Unlike GNNs that operate solely on graph‐structured inputs, *Hybrid Graph models* combine graph convolutional mechanisms with complementary architectures such as CNNs, transformers, or quantum neural layers, enabling simultaneous learning from molecular structures, multi‐omics profiles, and biological networks. For example, GraTransDRP [134] was proposed to improve drug representation and reduce data redundancy. GraTransDRP leveraged a Graph Transformer for more efficient drug feature extraction. Graph Transformers adapt the transformer architecture to graph‐structured data by using attention mechanisms to model relationships between nodes. Each node in the graph is associated with a feature vector that encodes its attributes. For cell line features, CNNs were used to learn from mutation, methylation, and transcriptomics data. Finally, the extracted drug and omics features were combined and fed into a fully connected network to predict drug response. To leverage gene pathway‐specific combinatorial implications, Yang et al. [135] introduced GPDRP (drug Graph and gene Pathway based Drug response prediction method), a novel multimodal DL model. In GPDRP, drugs were naturally represented by molecular graphs, while cell lines were described by gene pathway activity scores. The model separately learned features from these two data types using GNN with Graph Transformers for drugs and deep neural networks for gene pathways. To address the limitation of data collection, a novel hybrid quantum neural network [136] was proposed for drug response prediction, combining convolutional, graph convolutional, and deep quantum neural layers. This work represented a crucial step toward developing data‐efficient deep quantum algorithms with extensive quantum gates, offering a promising solution for personalized medicine problems where limited data availability is a critical constraint. Furthermore, SWnet [137] was introduced to predict drug response that integrates gene expression, genetic mutation, and chemical structure of compounds within a multi‐task convolutional architecture. Applied to the Genomics of Drug Sensitivity in Cancer (GDSC) and Cancer Cell Line Encyclopedia (CCLE) datasets, the model leveraged relevant cancer‐related genes (from oncology genetics databases and L1000 landmark genes) for genomic features, and cheminformatics features for compounds from PubChem or ChEMBL. By implementing an extended GNN for molecular graphs and a CNN for gene features, alongside multi‐tasking and self‐attention functions to capture compound similarity, this model outperformed recently published methods on the same datasets. These hybrid graph models enable the integration of heterogeneous biological and chemical modalities, allowing them to jointly model complex biological interactions between drugs, genes, and pathways. By leveraging graph structures for molecular representations and deep architectures for omics or pathway data, these models achieve richer feature extraction and enhanced generalization across diverse datasets. On the other hand, their architectural complexity increases both training time and computational cost, often requiring substantial resources for model optimization and hyperparameter tuning. The integration of diverse data types introduces challenges related to data harmonization and scaling, as differences in feature dimensionality or quality across omics and molecular datasets can degrade model performance.

*Knowledge‐Guided DL* integrates prior biological knowledge into neural network architectures to improve the prediction of complex biological outcomes, such as drug response. The DNN learns from both experimental data (e.g., gene expression, drug sensitivity) and structured biological knowledge, which can guide feature representation, reduce overfitting, and improve interpretability. For instance, Deep Neural Network Integrating Prior Knowledge (DIPK) [138], a DL framework that used self‐supervised techniques, was proposed to integrate multiple data types, including gene interaction relationships, gene expression profiles, and molecular topologies. Self‐supervised learning is a machine learning technique in which models are trained using automatically generated supervision signals derived directly from the data itself, rather than relying on manually annotated labels. Formulating auxiliary tasks, it enables models to learn meaningful and generalizable representations from large volumes of unlabeled data. DIPK demonstrated superior performance over existing methods on both known and novel cells and drugs, highlighting the critical role of gene interaction relationships in drug response prediction. Integrating drug‐target interaction information is intuitively valuable for predicting drug response. NetGP [139] was introduced to enhance DL‐based drug response prediction models by effectively utilizing drug target interaction. NetGP computed gene perturbation scores using a network propagation technique on a gene interaction network. It then ranked genes by these scores, feeding them into an MLP to generate a fixed‐dimension vector. This vector was integrated with existing drug response prediction models in a model‐agnostic way, allowing for broad applicability. Another important direction involves developing interpretable AI models that explicitly link molecular mechanisms to drug response. Jin and Nam [140] introduced a novel, interpretable AI model, HiDRA (hierarchical network for drug response prediction with attention), designed to predict drug responses in cancer cells at the gene, molecular pathway, and drug levels. Importantly, the model was confirmed to pay close attention to drug‐target genes and cancer‐related pathways during prediction, enhancing its interpretability. By incorporating prior biological knowledge, the model can potentially identify mechanistically meaningful patterns rather than correlations, improving interpretability and generalization. However, these frameworks require large, high‐quality, and well‐annotated datasets, which are often unavailable or inconsistent across drug responses, leading to potential overfitting and limited generalizability. It may also introduce bias or restrict model flexibility if the underlying biological knowledge is incomplete.

In summary, these computational frameworks represent a paradigm shift from empirical treatment selection toward data‐driven, personalized therapeutic strategies that can account for the unique molecular characteristics of both the drug and the patient's cancer (Table 6). A detailed overview of Graph‐based, Hybrid Graph, and Knowledge‐Guided DL approaches for drug response prediction is provided in Table 6. By explicitly modeling molecular topology and interaction patterns, *Graph‐based models* improved the interpretability of drug‐cell response prediction. These models are particularly suitable for scenarios where molecular structure and interaction topology are central to therapeutic response. However, their scalability is often limited by computational complexity and memory requirements, particularly when applied to large molecular graphs or high‐throughput screening datasets. *Hybrid Graph models* aim to simultaneously learn from heterogeneous biological data sources. These models are most advantageous when heterogeneous data modalities must be integrated, including molecular structures, multi‐omics profiles, and pathway‐level features. While these hybrid architectures enhance representational power and flexibility, they introduce substantial computational overhead and face challenges in data harmonization and model optimization across modalities. *Knowledge‐Guided DL* models are best applied in settings where reliable prior biological knowledge is available, such as curated interaction networks or well‐characterized signaling pathways. These methods incorporate prior biological knowledge, such as molecular interaction networks or pathway‐level information, into deep learning frameworks, which offer improved interpretability and alignment with known biological mechanisms. However, they may be prone to overfitting or limited generalizability when prior knowledge is incomplete or noisy. Accordingly, the optimal choice of model architecture depends on the availability of structural data, the need for multi‐modal integration, and the reliability of biological prior knowledge.

**TABLE 6:** A summary of the AI model developed for drug response prediction. Models are listed chronologically within model categories. The Key Features and Limitations column presents the advantages and disadvantages of each approach category in “A; B” format, where A represents the advantages and B represents the disadvantages.

Model type	Method	Key ideas	Year	References	Key features and limitations
Graph‐based models	GraphDRP	GCN	2022	Nguyen et al. [132]	Capture structural information; Struggle with scalability and computational efficiency
	GraphCDR	GNN	2022	Liu et al. [133]	
Hybrid Graph models	GraTransDRP	Graph Transformer + CNN	2023	Chu et al. [134]	Simultaneous learning from biological datasets; Struggle with computational cost and data harmonization
	GPDRP	GNN + Graph Transformer + Deep NN	2023	Yang et al. [135]	
	Hybrid Quantum NN	Convolutional + GCN + Quantum layers	2023	Sagingalieva et al. [136]	
	SWnet	Multi‐task CNN + Extended GNN	2021	Zuo et al. [137]	
Knowledge‐Guided DL	DIPK	Deep NN with self‐supervised learning	2024	Li et al. [138]	Capture complex biological dependencies; Potential overfitting and limited generalizability
	NetGP	Multi‐layer Perceptron + Network Propagation	2023	Pak et al. [139]	
	HiDRA	Hierarchical Network with Attention	2021	Jin and Nam [140]	

Abbreviations: GCN: Graph convolutional network; GNN: Graph neural network; CNN: Convolutional neural network.

### 4.3 Synergistic Treatment Prediction

The synergistic treatment is a therapeutic strategy using two or more drugs or interventions together where the combined effect is greater than the sum of their individual effects [141, 142]. While single‐agent immunotherapies have transformed cancer treatment, achieving durable responses in a broader patient population often necessitates the strategic combination of therapeutic agents. Synergistic treatment prediction in cancer immunotherapy leverages molecular and immune data to identify drug or intervention combinations whose combined effect exceeds that of individual treatments, enabling more effective and personalized therapy. In recent years, AI has been increasingly applied to predict synergistic treatment strategies in cancer immunotherapy, aiming to optimize patient outcomes by combining ICI with other therapeutic agents. These computational methods can be categorized as *Conventional ML*, *RL and optimization‐based models*, *Graph‐based models*, and *Multi‐omics DNN*.

*Conventional ML* has played an important role in predicting synergistic treatments. For example, the consensus machine learning‐driven prediction immunotherapy signatures (CMPIS) [145] using immune‐regulated genes and 303 different algorithms was developed. The optimal StepCox and Ridge algorithm comprising 16 key genes demonstrated superior prognostic performance. The results revealed that patients with lower CMPIS scores were more sensitive to immunotherapy and chemotherapy, while those with higher scores responded better to radiation therapy and EGFR‐targeted treatments. In relapsed/refractory (R/R) AML, Chen et al. [146] developed a systematic combinatorial design strategy that employs XGBoost to prioritize the most effective drug combinations for individual patients. Leveraging single‐cell transcriptomics and single‐agent response profiles from primary patient samples, this approach identified targeted combinations that co‐inhibit treatment‐resistant cancer cells. The research highlighted that dynamic changes in cell type composition between diagnostic and R/R stages necessitate personalized strategies to effectively target therapy‐resistant cells. Given the infeasibility of experimentally screening the vast number of possible drug combinations, computational models are crucial for prioritizing effective regimens. These *conventional ML* methods offer interpretability and stability, making them particularly effective for survival analysis and biomarker selection. However, these linear models are limited in capturing nonlinear and high‐order feature interactions inherent in complex immune responses.

Reinforcement learning (RL) is a machine learning technique where an autonomous agent learns to make sequential decisions by interacting with an environment to maximize a cumulative reward. Optimization‐based models aim to find the best solution to a problem under a set of constraints, often by maximizing or minimizing an objective function. *RL and optimization‐based models*, such as the deep reinforcement learning (DRL) framework integrated with ordinary differential equation (ODE)‐based tumor‐immune dynamics and Monte Carlo tree search algorithms, represent an important step toward treatment prediction. For example, Yao et al. [147] presented a framework combining DRL with an ODE‐based model of the TME to personalize ICI and chemotherapy strategies. DRL is a learning paradigm that combines RL with DNNs to solve sequential decision‐making problems. In DRL, an agent learns to make optimal decisions by interacting with an environment, receiving feedback in the form of rewards, and progressively improving its policy through trial‐and‐error. It optimizes long‐term outcomes by explicitly modeling actions, delayed rewards, and system dynamics. By integrating genomic and transcriptomic data, the researchers constructed an ODE‐based model capturing TME dynamics and trained a DRL agent to optimize treatment schedules. The personalized regimens generated by the DRL agent outperform fixed protocols, effectively tailoring therapy to tumor immune profiles, ranging from high‐dose chemotherapy for ‘cold’ tumors to combination strategies for ‘hot’ tumors. Similarly, Monte Carlo tree search optimization algorithms [148] were developed to optimize dosing patterns for anti‐vascular endothelial growth factor (VEGF) therapy combined with dendritic cells, significantly reducing drug doses and extending therapeutic windows. The *RL and optimization‐based models* can simulate and optimize complex, dynamic treatment schedules that account for temporal interactions between tumor evolution, immune response, and drug pharmacodynamics. However, such models rely heavily on accurate parameterization of biological systems, which is often constrained by incomplete or noisy clinical and molecular data.

*Graph‐based models* have recently demonstrated significant progress in predicting synergistic drug combinations. Specifically, AUarf et al. [149] developed piscesCSM, which leveraged graph‐based representations of small molecule chemical structures to accurately predict anticancer synergistic drug combinations against various cancer cell lines. The analysis of piscesCSM's interpretability revealed that simple physicochemical properties and graph‐based signatures are strong predictors of chemotherapy synergism. While current computational studies often focus on model architecture and molecular representation, the critical role of drug‐target information in combination therapies has been overlooked. To address this, the TAG‐CP network‐based framework [150] was developed to identify preclinical synergistic drug combinations by integrating drug‐target relationships into compound representation via a graph attention mechanism. In TAG‐CP, compounds were represented as nodes connected if they share common targets, and their molecular representations were learned by a modified attention‐based GNN. Compound‐compound pairs were then represented through an S‐kernel and concatenated with cancer cell line features as input for a DL model, with output calculated using the Bliss score from experimental data. Graph‐based representations of chemical structures provide an encoding of molecular topology that captures substructures, functional groups, and atom connectivity patterns, determining drug properties and interactions. It can learn hierarchical representations that identify which structural motifs contribute to synergy, potentially enabling rational drug design by highlighting pharmacophores associated with synergistic interactions. However, we might need to pay attention that drug combinations might be synergistic in vitro but clinically infeasible due to overlapping toxicities, incompatible administration routes or schedules, or antagonistic pharmacokinetic interactions. In addition, their performance is also highly sensitive to graph construction quality. Incomplete or biased interaction data can propagate noise and lead to inaccurate predictions.

*Multi‐omics DL* integrates diverse omics datasets to learn complex biological patterns, enabling accurate prediction of drug combinations. For example, AuDNNsynergy [151], was introduced to predict the synergy of pairwise drug combinations by integrating multi‐omics data (gene expression, copy number, and genetic mutation) from TCGA tumor samples. The encoded omics data, alongside the physicochemical features of individual drugs, serve as input for a DNN that predicts the synergy score of drug combinations against specific cancer cell lines. In addition, DeepMDS [152], a fully connected feed‐forward DNN approach that integrated multi‐omics data, was proposed to predict synergistic multi‐drug combinations within specific cell lines. DeepMDS was developed using a comprehensive dataset encompassing gene expression profiles of cancer cell lines, drug target information, and drug response data. Furthermore, the DD‐PRiSM (Decomposition of Drug‐Pair Response into Synergy and Monotherapy effect) DL pipeline [153] was developed to predict the effects of combination therapy. DD‐PRiSM employed a two‐model framework. The Monotherapy model predicted drug response curve parameters from drug structure and cell line gene expression to forecast cell viability, while the Combination therapy model assessed combination efficacy by analyzing individual drug effects and their synergistic interactions at specific dosages. These multi‐omics integration with DNN allows the models to capture complex biological dependencies and improve prediction accuracy for drug response and sensitivity. It also enables generalization across unseen drugs or tumor types and supports transfer learning. However, DL‐based frameworks generally require large, high‐quality, and well‐annotated datasets for reliable training. Scarce or inconsistent datasets can lead to overfitting or reduced model robustness when applied to new cohorts or unseen drugs. In addition, integrating multiple omics and network‐based features also increases computational cost and model complexity. This can make optimization challenging and reduce transparency, as deeper architectures become harder to interpret mechanistically.

These computational frameworks offer unprecedented opportunities to optimize treatment selection, dosing, and scheduling while minimizing toxicity and improving patient outcomes. As the field continues to evolve, the focus on personalized, data‐driven approaches promises to transform cancer treatment from empirical protocols to precisely tailored therapeutic strategies (Table 7). A detailed comparison of diverse synergistic treatment prediction methods is summarized in Table 7, categorized by model architecture into *Conventional ML*, *RL and optimization‐based models*, *Graph‐based models*, and *Multi‐omics DL*. *Conventional ML* is good at its interpretability, robustness, and relatively low computational cost. These methods are most appropriate for settings with limited sample sizes and strong requirements for interpretability. However, their capacity to model complex nonlinear interactions and dynamic treatment effects is limited compared with deep learning‐based frameworks. *RL and optimization‐based models* provide a principled way to optimize complex treatment schedules and drug interactions. These models are suitable for adaptive optimization problems, but their performance strongly depends on accurate biological modeling and parameterization, which may be difficult to obtain in clinical settings. *Graph‐based models* enhance the representation of molecular and relational features relevant to drug synergy, but their effectiveness is sensitive to the quality of graph construction and may be constrained by computational scalability. *Multi‐omics DL* models demonstrated a strong capacity to capture complex molecular interactions underlying synergistic effects. Nevertheless, they typically require large, well‐annotated datasets and may suffer from reduced interpretability and generalizability when data are limited or noisy. In conclusion, effective treatment strategy optimization requires aligning model choice with data availability, clinical objectives, and interpretability requirements.

**TABLE 7:** A summary of the AI model developed for synergistic treatment prediction. Models are listed chronologically within model categories. The Key Features and Limitations column presents the advantages and disadvantages of each approach category in “A; B” format, where A represents the advantages and B represents the disadvantages.

Model type	Method	Key ideas	Year	References	Key features and limitations
Conventional ML	CMPIS	Consensus ML	2024	Yin et al. [145]	Interpretability and stability; Limited flexibility for nonlinear interactions
	R/R AML Combinatorial Strategy	XGBoost	2025	Chen et al. [146]	
RL and optimization‐based models	DRL‐TIME Framework	DRL + ODE mathematical model	2024	Yao et al. [147]	Simulate and optimize complex interaction; Rely heavily on accurate parameterization of biological systems
	Monte Carlo VEGF Optimization	Monte Carlo tree search optimization	2019	Houy and Le Grand [148]	
Graph‐based models	piscesCSM	Graph‐based neural network	2024	AUarf et al. [149]	Capture representations of chemical and hierarchical structures; Sensitive to graph construction quality
	TAG‐CP	Graph Attention Network	2025	Zhang et al. [150]	
Multi‐omics DL	AuDNNsynergy	DNN	2021	Zhang et al. [151]	Capture complex molecular interactions; Requires large, high‐quality datasets.
	DeepMDS	Fully connected feed‐forward DNN	2022	She et al. [152]	
	DD‐PRiSM	Dual‐model DL pipeline	2024	Jin et al. [153]	

Abbreviations: AML: Acute myeloid leukemia; DRL: Deep reinforcement learning.

## 5 Foundation Models and LLM for Cancer Immunotherapy

**FIGURE 5:**
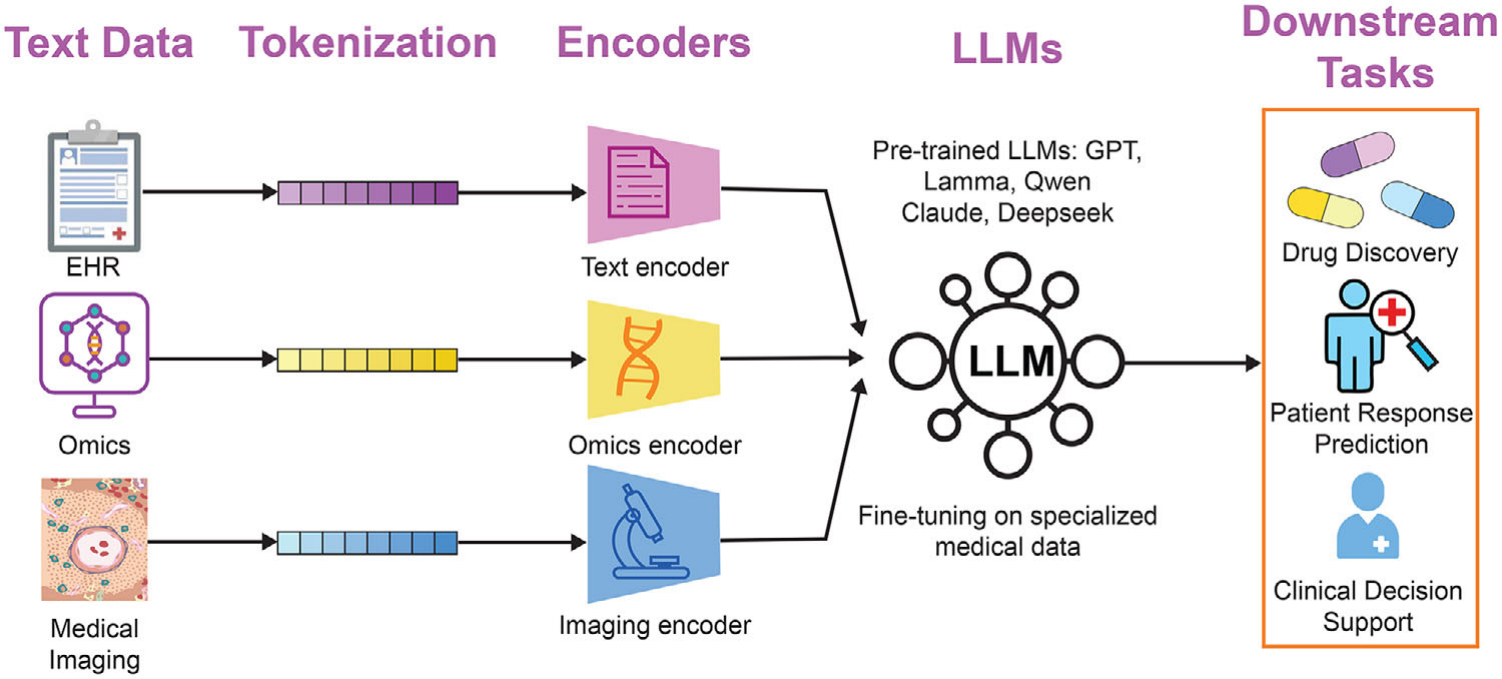
Overview of LLM applications for cancer immunotherapy. Heterogeneous biomedical data, including electronic health records, omics data, and medical imaging, are processed through tokenization and encoding modules. These modality‐specific embeddings are jointly leveraged to fine‐tune pre‐trained large language models, such as GPT [157], LLaMA [158], Qwen [159], Claude [160], and DeepSeek [161], enabling downstream applications in drug discovery, treatment response prediction, and clinical decision support.

The rapid advancements in AI, particularly the emergence of foundation models and LLMs, have introduced transformative capabilities for cancer immunotherapy. Foundation models are pre‐trained on large‐scale datasets (e.g., text, images, audio, etc.) to support a wide range of downstream tasks. Through self‐supervised learning or weakly supervised learning, these models can acquire representations that capture complex patterns and relationships within data, enabling improved performance across diverse applications [154, 155]. This transition from narrowly specialized algorithms to adaptable, general‐purpose models defines the foundation model paradigm. LLMs constitute a prominent category of foundation models trained to model sequential data using language modeling objectives. While originally developed for NLP, LLMs have been successfully extended to biological and clinical domains by treating biological sequences (e.g., DNA, RNA, proteins, peptides) and structured medical text (e.g., clinical notes, pathology reports, and biomedical literature) as languages [156]. Through large‐scale pre‐training, LLMs learn contextualized representations that encode long‐range dependencies, semantic relationships, and hierarchical structures, enabling them to perform reasoning, prediction, and generation tasks across diverse biomedical applications. These models, built on transformer architectures and trained on vast datasets encompassing biological, clinical, imaging, and scientific literature, possess the remarkable ability to identify intricate patterns and relationships within complex biological information. Figure 5 provides an overview of LLMs for cancer immunotherapy applications. Specifically, heterogeneous data (e.g., EHR, omics profiles, and medical imaging) are tokenized and encoded through modality‐specific encoders. The encoded representations from different modalities are integrated into fine‐tuned LLMs, where pre‐trained foundation models that have learned general patterns from massive datasets are adapted through fine‐tuning on specialized biomedical data. The fine‐tuned LLMs can then be deployed for various downstream applications, including drug discovery, treatment response prediction, and clinical decision support. Specifically, there are three main types of LLM for cancer immunotherapy: *Generalist LLMs*, *Specialized LLMs*, and *Multimodal foundation models*.

### 5.1 Generalist LLMs

The *Generalist LLMs* serve as a multi‐domain, multi‐task foundation model capable of performing diverse therapeutic reasoning and prediction tasks across molecules, proteins, and diseases with minimal fine‐tuning. These models are not originally designed for cancer immunotherapy applications. Rather, they are pre‐trained on large‐scale, heterogeneous datasets spanning multiple domains, enabling them to learn generalizable representations and reasoning capabilities. Through subsequent task‐specific fine‐tuning or prompt‐based adaptation, these broadly trained models can be effectively repurposed for cancer immunotherapy tasks. These models provide a broad knowledge base and versatile reasoning capabilities, making them suitable for applications in cancer immunotherapy. For example, Tx‐LLM and TxGemma have demonstrated superior performance across 66 drug discovery tasks across chemical and biological modalities. Tx‐LLM [162], fine‐tuned from PaLM‐2, can predict diverse therapeutic properties by processing molecular structures alongside clinical or biological context. TxGemma [163] is a suite of generalist LLMs fine‐tuned from Gemma‐2 on a comprehensive dataset (small molecules, proteins, nucleic acids, diseases, and cell lines) for broad therapeutic property prediction and scientific reasoning across molecules, proteins, and diseases. It required less data for fine‐tuning, making it suitable for clinical applications with limited datasets. The practical applications include GPT models achieving clinically relevant accuracy in predicting immunotherapy response in HCC and automating the identification of irAEs. Xu et al. [164] evaluated the performance of LLMs, including GPT‐4, GPT‐4o, and Gemini, in predicting immunotherapy response in unresectable HCC compared to physicians of varying expertise levels based on clinical and CT images. Bejan et al. [165] investigated the capabilities of GPT models (GPT‐3.5, GPT‐4, and GPT‐4o) in identifying irAEs from unstructured patient notes in healthcare datasets. The model performance was evaluated on manually annotated data from 442 patients across electronic health records and clinical trials who received ICI therapy. These *Generalist LLMs* enable cross‐domain generalization by learning universal representations from large‐scale, heterogeneous datasets, which allows them to perform diverse downstream tasks such as drug‐target interaction prediction, disease classification, and literature‐based hypothesis generation without task‐specific retraining. However, domain mismatch and bias can arise when foundation models trained on general or non‐biomedical data are transferred to specialized biological tasks, potentially reducing accuracy or introducing misleading associations.

### 5.2 Specialized LLMs

The *Specialized LLMs* are LLMs that are fine‐tuned or pre‐trained on domain‐specific data to excel at tasks within a particular field. They are tailored for deep expertise in a focused area, improving accuracy, relevance, and interpretability for domain‐specific applications. In the realm of immune system modeling, tcrLM [166], a lightweight protein language model, was developed to predict T cell receptor‐antigen binding with superior generalizability from TCR sequences, while TULIP [167] applied transformer architecture from language models within an unsupervised learning framework to recognize the specific TCRs binding an epitope. TULIP enabled the integration of all available data sources regardless of their quality or completeness, while avoiding the sampling bias inherent in supervised approaches that rely on artificially selected negative examples. For other molecular‐level approaches, specialized models like pMHChat and HLApollo have advanced our understanding of MHC‐peptide interactions through integration of LLMs and deep hypergraph learning, and transformer‐based architectures, respectively. The pMHChat [168] was proposed to predict MHC class II‐peptide binding interactions based on MHC pseudo‐sequences and peptide sequences, addressing the critical need for understanding these interactions in immune system research with applications in neoantigen design, vaccine development, and personalized immunotherapy. HLApollo [169] leveraged the language of peptides, MHC, and source proteins to predict peptide‐MHC‐I (pMHC‐I) presentation through an end‐to‐end approach that handles MHC‐I sequences and deconvolves multi‐allelic data, while employing a negative‐set switching strategy to address the challenge of misassigned negatives in unlabeled ligandome data. Similarly, Eom et al. [170] presented a computational approach combining homology searches and protein language models to discover and prioritize enzymes based on their kinetic parameters, specifically targeting kynureninases for cancer therapy applications. The researchers focused on identifying kynureninases that could hydrolyze L‐kynurenine, an immunosuppressive metabolite that contributes to tumor immune evasion, to enhance anticancer therapy by overcoming the immunosuppressive TME. In addition, DeepNeoAG [171] was introduced to precisely predict neoantigens via innovatively combining global sequence‐level information captured by a pre‐trained protein language model with local sequence‐based features extracted by a multi‐window scanning CNN. By focusing on domain‐specific biological and molecular data, these *Specialized LLMs* achieve enhanced biological interpretability and contextual relevance compared to *Generalist LLMs*. These models exhibit superior generalization and transferability across related biological tasks, such as epitope prediction, antigen presentation, and enzyme characterization, by leveraging pre‐training on large‐scale protein or genomic datasets. However, domain specialization limits general applicability because models trained on narrow biological contexts may underperform when transferred to other immunological or molecular systems.

### 5.3 Multimodal Foundation LLMs

The *Multimodal foundation LLMs* are LLMs that integrate information from multi‐omics datasets, medical imaging, and textual sources to provide personalized predictions of drug response and immunotherapy efficacy. For instance, UniCure [172], the pre‐trained foundation model integrating both omics and chemical foundation models (UCE and Uni‐mol), was produced to predict transcriptomic responses to drugs across diverse cellular and tissue contexts. Using parameter‐efficient fine‐tuning, UniCure was trained on over 1.8 million perturbation RNA‐seq profiles over 22,000 compounds, 166 cell types, and 24 tissues. Fine‐tuned on patient‐derived tumor‐like clusters and real‐world data, UniCure generated individualized therapeutic predictions on patients’ tissue samples. In addition, it enabled individualized drug prioritization, patient stratification, and response prediction, which offered a biologically grounded tool to advance personalized precision oncology and accelerate drug discovery. Similarly, MUSK (Multimodal transformer with Unified maSKed modeling) [38], a vision‐language foundation model, was designed to integrate multimodal clinical data by leveraging large‐scale, unlabeled, and unpaired pathology images and text data to address the challenge of scarce well‐annotated multimodal datasets in clinical settings. MUSK demonstrated superior performance across 23 patch‐level and slide‐level benchmarks, including image‐to‐text and text‐to‐image retrieval, visual question answering, image classification, and molecular biomarker prediction. In addition, the model exhibited strong capabilities in outcome prediction tasks such as melanoma relapse prediction, pan‐cancer prognosis prediction, and immunotherapy response prediction in lung and gastro‐esophageal cancers. By learning shared representations across modalities, the multimodal foundation models can capture complementary biological information, leading to more accurate and biological predictions. It also helps to uncover relationships between molecular, cellular, and phenotypic layers of disease. Despite their promise, multimodal foundation models face practical challenges that data heterogeneity and imbalance across modalities complicate model training and can lead to biased or modality‐dominant representations. In addition, integration and alignment of modalities, especially those differing in dimensionality, resolution, and noise, often require sophisticated architecture or large‐scale curated datasets.

In summary, these foundation models and LLM provide a powerful, generalizable framework to enhance therapeutic development, improve clinical decision‐making, and accelerate the discovery of personalized cancer treatments (Table 8). Comprehensive comparisons of various strategies for cancer immunotherapy are presented in Table 8. *Generalist LLMs* provide strong versatility and broad reasoning capabilities; however, their performance in specialized cancer immunotherapy tasks may be affected by domain mismatch, bias, and limited biological grounding. *Specialized LLMs* enhance biological interpretability and contextual relevance in immune‐related predictions. However, their specialization often constrains general applicability, and models trained on narrow biological contexts may not readily transfer to other immunological or clinical settings. *Multimodal foundation LLMs* can capture complementary biological information across molecular, cellular, and phenotypic layers, enabling more personalized and biologically informed predictions. Nevertheless, challenges remain in handling data heterogeneity, modality imbalance, and the substantial computational and data requirements needed for effective multimodal alignment. Consequently, selecting an appropriate foundation model requires careful consideration of task complexity, data modality availability, and the balance between generalizability and biological specificity.

**TABLE 8:** A summary of the foundation model and LLM developed for cancer immunotherapy. Models are listed chronologically within model categories. The Key Features and Limitations column presents the advantages and disadvantages of each approach category in “A; B” format, where A represents the advantages and B represents the disadvantages.

Model type	Method	Key ideas	Year	References	Key features and limitations
Generalist LLMs	Tx‐LLM	Large language model fine‐tuned from PaLM‐2	2024	Chaves et al. [162]	Cross‐domain generalization; Potential domain mismatch and bias
	TxGemma	Suite of generalist LLMs fine‐tuned for therapeutics	2025	Wang et al. [163]	
	GPT Models	GPT‐4, GPT‐4o, Gemini	2025	Xu et al. [164]	
	irAE‐GPT	GPT‐3.5, GPT‐4, GPT‐4o	2025	Bejan et al. [165]	
Specialized LLMs	tcrLM	Masked language model for TCR sequences	2024	Fang et al. [166]	Enhanced biological interpretability and contextual relevance; Limited general applicability
	TULIP	Transformer‐based unsupervised language model	2024	Meynard‐Piganeau et al. [167]	
	pMHChat	LLMs + deep hypergraph learning	2025	Ma et al. [168]	
	HLApollo	Transformer‐based model	2024	Thrift et al. [169]	
	Kynureninase Discovery	Protein language models + homology searches	2025	Eom et al. [170]	
	DeepNeoAG	Protein language models + CNN	2024	Chuang et al. [171]	
Multimodal foundation models	UniCure	Foundation model integrating omics and chemical models (UCE and Uni‐mol)	2025	Chen et al. [172]	Capture complementary biological information; Hard to deal with data heterogeneity and imbalance across modalities.
	MUSK	Vision‐language foundation model with unified masked modeling	2025	Xiang et al. [38]	

Abbreviations: TCR, T cell receptor; CNN, convolutional neural network.

## 6 Challenges and Future Directions

Recent decades have witnessed the widespread use of leveraging AI into cancer immunotherapy to improve the precision of outcome prediction, personalize treatments, and enhance clinical applicability across major immunotherapy modalities, including ICIs, personalized neoantigen vaccines, and adoptive cell therapies such as CAR‐T. Although the use of AI/ML in cancer immunotherapy has been widely explored, there are various challenges and limitations. These include both technical and regulatory issues. Specifically, there are fourteen types of challenges and limitations, namely *heterogeneity and integration*, *small dataset*, *biomarker reproducibility*, *model interpretability*, *missing value*, *computational source requirement*, *data privacy*, *requirement of expert intervention*, *regulatory and validation*, *causal inference models*, *uncertainty quantification*, *model fairness/bias*, *prospective validation*, and *benchmark datasets scarcity*.

### 6.1 AI for Patient Stratification in Cancer Immunotherapy

AI‐enabled patient stratification includes tumor subtype classification and risk modeling for response, relapse, and survival, with the overarching goal of achieving robust generalization across cohorts and clinical settings. This category of approaches is primarily limited by challenges like *heterogeneity and integration*, *causal inference models*, *uncertainty quantification*, and *model fairness/bias*. In practice, model performance is often constrained by cross‐platform and cross‐site heterogeneity, including batch effects, inconsistent preprocessing workflows, and differences in cohort composition. These limitations are frequently coupled with clinical confounding because patient management differs across cohorts in treatment history and line of therapy, making correlation‐driven models harder to translate into actionable decisions. For safe deployment, models should quantify prediction uncertainty under distribution shift and routinely evaluate subgroup performance to detect disparities linked to demographic or geographic imbalance.

#### 6.1.1 Heterogeneity and Integration

The mechanisms of cancer immunotherapy have been revealed by the rapid development of multi‐omics technologies; However, the integration and analysis of large‐scale multi‐omics data still pose considerable challenges. The presence of batch effects is one of the major issues arising from heterogeneous data acquisition platforms, which can obscure true biological signals. A widely used solution is representation‐level harmonization prior to downstream modeling, where joint dimensionality reduction methods (e.g., PCA, NMF, and CCA) project samples from different cohorts or modalities into a shared latent space that preserves biological variation while reducing batch‐specific components [173]. By integrating data modalities like genomics and medical imaging [174, 175], multimodal ML models enhance the capacity to extract comprehensive insights, thereby supporting more accurate prediction and interpretation of immunotherapy response. Effectiveness of these approaches should be evaluated through cross‐cohort validation and by assessing whether the learned latent space reduces batch dependence while retaining biologically meaningful immune patterns.

#### 6.1.2 Causal Inference Models

Although most current AI studies in immuno‐oncology focus on predictive performance, clinical decision‐making often requires causal understanding (i.e., whether a factor is truly driving response or merely correlated due to confounding). Causal‐inference‐aware study designs and models can strengthen clinical interpretability, including target trial emulation for observational cohorts, propensity‐based adjustment or doubly robust estimators, and causal representation learning when multi‐omics and clinical covariates are jointly modeled. This typically starts by explicitly defining the treatment, outcome, and confounders, followed by sensitivity analyses for unmeasured confounding, and reporting treatment‐effect estimates alongside predictive metrics, enabling more clinically actionable insights beyond association‐based prediction.

#### 6.1.3 Uncertainty Quantification

For high‐stakes immunotherapy decisions, point predictions alone are insufficient; On the contrary, models should quantify uncertainty to support risk‐aware deployment. Approaches for uncertainty quantification include probabilistic calibration, ensemble‐based uncertainty, Bayesian or approximate Bayesian methods, and conformal prediction to provide well‐calibrated confidence measures. Uncertainty should be evaluated using calibration curves and decision‐relevant metrics (i.e., selective prediction/abstention where the model defers uncertain cases to clinicians), ensuring that uncertainty estimates are meaningful and improve safety rather than merely adding complexity.

#### 6.1.4 Model Fairness/Bias

Model performance can vary systematically across demographic groups, tumor subtypes, and institutions, raising concerns about fairness and potential harm if models amplify existing healthcare disparities. This can be addressed by embedding subgroup‐aware evaluation into model development, including routine reporting of performance stratified by key attributes (e.g., sex, age, ancestry, site), explicit testing for cross‐center distribution shift, and applying mitigation strategies such as reweighting, domain generalization, or fairness‐constrained learning when disparities are detected. Importantly, fairness assessments should be tied to clinically meaningful endpoints and transparently documented to support responsible translation.

### 6.2 AI for Biomarker Identification in Cancer Immunotherapy

Biomarker identification covers transcriptomics, epigenomics, proteomics, pathomics, genomics, EHR, and multi‐omics settings, aiming to discover predictive and prognostic signals that remain biologically credible and reproducible across cohorts, modalities, and clinical contexts. This category of approaches is primarily impacted by challenges like *biomarker reproducibility*, *model interpretability*, *missing values*, and the *requirement of expert intervention*. In practice, biomarker reproducibility is often limited because signatures derived from a single cohort may not be found and validated in independent datasets, largely due to platform differences and cohort‐specific feature selection. These issues become more severe in multi‐omics studies, where missing modalities and uneven sampling complicate integration. Pathomics and EHR‐based biomarkers introduce additional constraints that affect real‐world scalability, including site‐dependent image variation, expert‐dependent annotation and quality control, and structured or unstructured missingness in clinical records. Reliable biomarker discovery will require standardized data construction and multi‐cohort validation, paired with methods that emphasize stability and interpretability.

#### 6.2.1 Biomarker Reproducibility

Many ML models have been constructed for immunotherapy response prediction, which, however, may show limited generalizability and perform suboptimally on unseen datasets. This is partly due to inconsistencies in marker gene selection across studies, as traditional statistical or ML‐based feature selection often yields dataset‐specific results. Transfer learning offers a feasible way to improve robustness by pretraining models on large related resources, such as pan‐cancer transcriptomic or immunogenomic cohorts, and then fine‐tuning on smaller immunotherapy‐specific datasets to adapt to task‐specific endpoints and distributions [176, 177, 178]. By leveraging shared immune patterns across datasets, transfer learning reduces sensitivity to cohort‐specific feature selection and improves model robustness and training efficiency. To ensure reproducibility, such models should be evaluated through cross‐cohort validation and benchmarked against established immunotherapy biomarkers, enabling consistent performance assessment across independent datasets.

#### 6.2.2 Model Interpretability

Additionally, the opaque nature of many ML models, often described as “black boxes” [179], poses a challenge for biological interpretation, which in turn reduces clinical trust, scientific insight, and interpretability. Physicians are reluctant to rely on predictions from AI models that cannot be explained or validated against known pathological or biological processes. This limitation can be mitigated by incorporating explainable artificial intelligence (XAI) techniques into the modeling workflow to elucidate both global and local drivers of model predictions [180]. For example, post‐hoc interpretability methods such as SHapley Additive exPlanations (SHAP) [181] values can be applied to quantify both global and sample‐specific feature contributions, helping link model outputs to interpretable signals such as immune infiltration patterns, pathway activities, or spatially informative regions in histopathology images [182].

#### 6.2.3 Missing Value

Another important challenge in working with biomedical datasets is the presence of missing values. While one of the conventional solutions is to exclude patients with incomplete data, this can lead to selection bias, particularly if the missingness is systematically related to clinical or molecular variables [183]. Another standard mitigation way is to incorporate imputation into preprocessing, for example, using multiple imputation to estimate missing values while propagating uncertainty [184]. To avoid information leakage, imputation models should be fit on the training set only and then applied to validation/test cohorts. The downstream impact should be evaluated by comparing model performance and feature stability with and without imputation to ensure conclusions are not driven by the missing‐data mechanism [185].

#### 6.2.4 Requirement of Expert Intervention

Advancing immuno‐oncology requires the integration of diverse expertise across computational, biological, and clinical domains. However, interdisciplinary work can become a bottleneck when terminologies and validation standards differ across teams, leading to inconsistent ground truth and delayed translation. A feasible solution is to formalize structured interdisciplinary workflows with clearly defined roles and handoffs across the pipeline, spanning data curation and harmonization, model development, interpretability review, experimental validation, and clinical feasibility assessment. In practice, this is typically supported by regular cross‐functional review meetings, sharing data dictionaries/annotation standards, pre‐specified analysis plans, and evaluation protocols aligned with clinically meaningful endpoints. To further promote data sharing and team‐based discovery, national agencies such as the NIH and NCI have launched initiatives like the Immuno‐Oncology Translational Network (IOTN) [186] and the Cancer Center Support Grants. Notably, the NIH Data Management and Sharing (DMS) policy [187] mandates public availability of research data, fostering cross‐disciplinary transparency and accelerating the development of novel tools and insights.

### 6.3 AI for Treatment Strategy Optimization in Cancer Immunotherapy

Treatment strategy covers neoantigen identification, drug response prediction, and synergistic treatment. It aims to generate actionable recommendations that can influence therapy selection. This category of approaches is primarily affected by challenges like *small datasets*, *benchmarking datasets scarcity*, and *prospective validation*. These tasks are often limited by the scarcity of high‐quality labels. Experimentally validated neoantigen immunogenicity is difficult and costly to obtain, and clinically grounded outcomes for drug response and combination benefit are also sparsely available. As a result, training cohorts are typically small and evaluation benchmarks remain fragmented, which complicates fair comparison and slows reproducible progress. Because these models directly inform therapy selection, prospective and preferably multi‐center validation is essential to confirm clinical benefit under real‐world workflows and to avoid optimistic performance estimates from retrospective cohorts.

AI‐enhanced organoid‐based immunotherapy modeling and engineered T‐cell therapies (e.g., CAR‐T, TCR‐T) are rapidly advancing, but they also highlight key gaps in data standardization and generalization. Patient‐derived tumor organoids and organoid‐immune co‐cultures provide experimentally tractable systems to study immune infiltration, activation, cytotoxicity, and immune evasion under controlled perturbations, offering a bridge between simplified 2D assays and in vivo models [188]. Tumor organoid‐on‐a‐chip platforms further extend this by incorporating tissue‐like architecture and perfusion to better emulate tumor‐immune interactions and evaluate immunotherapy efficacy [189]. As these systems increasingly couple high‐content imaging with molecular profiling, AI can help connect morphology and microenvironmental states to functional readouts and treatment response, enabling scalable mechanism and biomarker discovery [190]. In parallel, CAR‐T/TCR‐T development generates complex datasets spanning receptor/epitope specificity, circuit and costimulatory design, and clinical efficacy/toxicity. AI models for TCR‐epitope prediction are maturing and can support specificity and cross‐reactivity assessment, a central safety/efficacy bottleneck for TCR‐T. Finally, integrating CRISPR perturbation screens with single‐cell phenotyping offers a systematic way to identify genetic interventions that improve engineered T‐cell fitness, persistence, and function, especially under suppressive TME. Large‐scale CRISPR screens have mapped regulators of T‐cell exhaustion and persistence, providing mechanistic targets that can be prioritized for engineering [191]. Recent CAR‐T–specific platforms now combine genome‐wide perturbations with clinically motivated functional readouts and pooled in vivo single‐cell validation, enabling the discovery of edits that enhance proliferation while reducing exhaustion [192]. Together, these perturbations combined frameworks shift CAR/TCR optimization toward an engineering cycle that is more data‐driven and generalizable [193].

#### 6.3.1 Small Dataset

Immunotherapy has been regarded as a promising strategy for cancer treatment, prompting a surge in global clinical trials. However, the majority of clinical trials have concentrated on PD‐1/PD‐L1 blockade [16]. The small size of the dataset presents a major challenge for developing robust ML/DL models, particularly models that require large‐scale training data to prevent overfitting and ensure generalizability [194]. To overcome this limitation, researchers have explored the use of synthetic data augmentation through advanced generative models. Techniques such as GANs [195] and diffusion models [196] are increasingly being applied to biomedical data for data augmentation, such as histopathology images [197, 198] and omics profiles [199], thereby expanding the effective training set. Because synthetic augmentation can introduce artifacts or amplify biases, its value should be demonstrated by improved downstream performance and consistent results under external validation rather than by visual plausibility alone.

#### 6.3.2 Benchmark Datasets Scarcity

The scarcity of well‐curated benchmark datasets with standardized endpoints, data splits, and reporting practices remains a major barrier to reproducibility and fair comparison across studies. A suitable pathway is to promote community benchmarks for key immunotherapy tasks (e.g., response prediction, survival, toxicity, biomarker discovery) with unified evaluation protocols, external test sets, and transparent reporting of preprocessing and cohort selection. When public data are limited, federated or privacy‐preserving benchmarking frameworks can enable cross‐institutional evaluation without sharing raw patient data, improving generalizability assessment and accelerating progress.

#### 6.3.3 Prospective Validation

Many published AI models are validated retrospectively on curated datasets, whereas clinical adoption typically requires prospective evaluation under real‐world workflows. Actionable steps include designing multi‐center prospective studies with pre‐specified endpoints and comparators, integrating the model into clinical pathways with clear intended use, and conducting implementation‐focused evaluations (e.g., impact on decision consistency, time‐to‐treatment, or patient outcomes). Post‐deployment monitoring plans are also essential to detect performance drift and maintain safety as patient populations and clinical practices evolve.

### 6.4 Foundation Models and LLM for Cancer Immunotherapy

Foundation models and LLMs are increasingly used in immuno‐oncology to support knowledge‐intensive workflows such as feature extraction and clinical summarization. This category of approaches is primarily constrained by challenges like *data privacy*, *regulatory and validation*, and *computational source requirements*. Data governance is a primary constraint because training and fine‐tuning often require sensitive clinical text, images, and multi‐omics data distributed across institutions. In addition, foundation models are expensive to train and adapt, often requiring specialized hardware and carefully engineered deployment pipelines, which can limit reproducibility and slow clinical translation. For clinical deployment, it should include evaluation plans for real‐workflow testing, safety monitoring, and drift surveillance as practice patterns evolve. Progress in this area, therefore, depends on regulatory‐ready validation packages, including reproducible documentation, controlled model updates, and post‐deployment monitoring, rather than focusing solely on scaling model size.

#### 6.4.1 Data Privacy

AI‐driven cancer research increasingly relies on large volumes of sensitive patient data, making data privacy and security critical concerns. Traditional centralized data aggregation approaches raise substantial risks related to confidentiality, regulatory compliance, and patient trust. Federated learning [200] addresses this by enabling collaborative training across institutions while keeping raw patient data local, i.e., only model updates (e.g., parameters or gradients) are shared, allowing data to remain within each institution's firewall and improving generalizability across diverse populations. To further reduce leakage risk from shared updates, federated training is often combined with privacy‐enhancing mechanisms such as secure aggregation/encryption and, when appropriate, differential privacy. Blockchain technology [201] can complement these workflows by providing auditable provenance and access control via an immutable ledger of data/model transactions, improving integrity and accountability in multi‐party collaborations. Together, federated learning and blockchain support decentralized AI development with stronger privacy guarantees and traceability.

#### 6.4.2 Computational Source Requirement

AI‐based research, particularly in immuno‐oncology, is computationally intensive. DL models often involve millions to billions of parameters, requiring substantial memory and processing power. As a result, training such models can be computationally expensive and sometimes infeasible. Limited access to such high‐performance hardware can pose a significant barrier, especially for smaller institutions or research teams with constrained resources. Cloud computing [202] and distributed computing technologies [203] mitigate this barrier by providing on‐demand access to specialized accelerators, including scalable graphics processing unit (GPU) or tensor processing unit (TPU) clusters that support elastic training without large upfront infrastructure investment. Standardized containerized environments and reproducible job configurations further help ensure that experiments can be rerun consistently across institutions and over time.

#### 6.4.3 Regulatory and Validation

The integration of AI into clinical practice, particularly in cancer immunotherapy, is hindered by complex regulatory and validation hurdles. Agencies such as the FDA and EMA require AI tools to meet rigorous standards for approval as Software as a Medical Device (SaMD), including compliance with frameworks like ISO 13485 and IEC 62304 [204], as well as validation through prospective clinical trials or multi‐center studies. These processes are time‐consuming and resource‐intensive. Alignment is strengthened when development begins with a clearly defined intended use and clinical context, prespecified endpoints and comparator standards, and cross‐site validation designed to reflect real‐world heterogeneity, supported by reproducible preprocessing, traceable documentation, and version‐controlled model updates. Deployment also depends on institutional readiness, including interoperable data pipelines, clinician‐facing workflows, and post‐deployment monitoring to detect drift and maintain safety. Together, clearer guidance and structured validation‐and‐operations frameworks can substantially accelerate trustworthy adoption of AI in immuno‐oncology.

Detailed information of challenges and opportunities for AI application in cancer immunotherapy are summarized in Table 9, where challenges (including *heterogeneity and integration*, *small dataset*, *biomarker reproducibility*, *model interpretability*, *missing value*, *computational source requirement*, *data privacy*, *requirement of expert intervention*, *regulatory and validation*, *causal inference models*, *uncertainty quantification*, *model fairness/bias*, *prospective validation*, and *benchmark datasets scarcity*), negative impacts, actionable solutions are elaborated.

**TABLE 9:** A summary of challenges and opportunities for future AI development for cancer immunotherapy. The table summarizes key challenges limiting AI applications in cancer immunotherapy, while outlining corresponding opportunities such as multi‐omics integration, explainable and causal modeling to advance reliable and clinically actionable AI solutions.

Challenge	Negative impact	Actionable solution
Heterogeneity and integration	Difficulty in integrating datasets	Joint dimension reduction algorithms
Small dataset	Model overfitting	Data augmentation
Biomarker reproducibility	Difficulty in validating biomarkers	Transfer learning algorithms
Model interpretability	Reduced trust in AI models	Explainable AI methods
Missing value	Bias and uncertainty	Improved imputation methods
Computational source requirement	Computationally costly or infeasible	Cloud computing, specialized AI hardware
Data privacy	Ethical and legal risks	Federated learning
Requirement of expert intervention	Complex communication and integration issues	Structured interdisciplinary teams
Regulatory and validation	Slow adoption	Clearer regulatory guidelines
Causal inference models	Non‐causal conclusions	Target trial emulation; causal adjustment
Uncertainty quantification	Overconfident, unsafe predictions	Calibration; conformal prediction
Model fairness/bias	Subgroup performance disparities	Subgroup auditing: bias mitigation
Prospective validation	Poor real‐world generalization	Prospective multi‐center trials
Benchmark datasets scarcity	Limited comparability, reproducibility	Standardized benchmarks; shared protocols

## 7 Conclusion

AI/ML models are reshaping cancer immunotherapy from proof‐of‐concept experiments to large‐scale and accessible clinical applications that can illuminate tumor complexity, predict responses, and guide treatment design [205]. Across the landscape reviewed here, AI has achieved several significant advances in cancer immunotherapy. First, patient stratification has been reshaped by advanced AI that integrates pathology, multi‐layer omics, and clinical variables into unified embeddings, capturing hidden lineages and survival trajectories. Toward a clinical application of an AI model, the next step is to widen representation, standardize, and automate mapping processes for an equally robust molecular identity [206]. Second, AI‐driven biomarker identification now encompasses cross‐layer signatures distilled from genomics, epigenetics, transcriptomics, proteomics, radiomics, and spatial context [207]. Traditional ML models such as SVM, XGBoost, and NMF enable interpretable feature selection, while DL architecture including transformers and GAN extract non‐linear molecular and spatial patterns from high‐dimensional data. Language models further integrate biomedical information to facilitate knowledge‐driven biomarker reasoning. The immediate challenge is to validate these composite signatures prospectively, translate them into economical assays that run on minimal tissue or plasma for diagnostics [205]. Parallel efforts linking serial imaging with circulating markers will build “digital biopsies” that track spatial heterogeneity and evolution, reducing the need for repeat sampling [208]. Third, AI‐driven treatment strategy optimization addresses the complexity of therapeutic decision‐making across multiple dimensions. By integrating multi‐omics profiles, imaging data, and HER through DL architectures [205, 209], AI models enable neoantigen identification for personalized vaccine design, predict patient‐specific drug response to guide therapeutic selection, and identify synergistic treatment combinations that overcome resistance mechanisms. Finally, foundation‐scale models signal a shift from isolated predictors to holistic patient representations [210]. Their abilities to generalize across disease and treatment types could interpret routine sequencing, flag actionable vulnerabilities, and suggest rational combination strategies at the bedside. The challenges of developing advanced AI for cancer immunotherapy are comprehensively discussed. Translating these advances into clinical practice requires addressing critical technical barriers such as data integration and model generalizability, the need for interpretable and reproducible biomarkers, privacy‐preserving frameworks for multi‐institutional collaboration, and regulatory pathways that validate AI tools efficiently without compromising rigor. Overcoming these obstacles needs interdisciplinary partnerships and institutional support, which enables the transformation of AI‐based cancer immunotherapy from promising research into practical clinical solutions, supporting clinicians and empowering patients.

## Funding

Research reported in this publication was supported by the U.S. National Science Foundation under Award Number 2500836, the Office of the Director, National Institutes of Health of the National Institutes of Health under Award Number R03OD038391, and by the National Cancer Institute of the National Institutes of Health under Award Number P30CA036727. This work was supported by the American Cancer Society under award number IRG‐22‐146‐07‐IRG, and by the Buffett Cancer Center, which is supported by the National Cancer Institute under award number CA036727. This work was also partially supported by the National Institute of General Medical Sciences under Award Numbers P20GM103427. This study was in part financially supported by the Child Health Research Institute at UNMC/Children's Nebraska. This work was also partially supported by the University of Nebraska Collaboration Initiative Grant from the Nebraska Research Initiative (NRI). The content is solely the responsibility of the authors and does not necessarily represent the official views of the funding organizations.

## Conflicts of Interest

The authors declare no conflicts of interest.
